# Lipidomic profiling reveals biosynthetic relationships between phospholipids and diacylglycerol ethers in the deep-sea soft coral *Paragorgia arborea*

**DOI:** 10.1038/s41598-021-00876-5

**Published:** 2021-10-28

**Authors:** Andrey B. Imbs, Peter V. Velansky

**Affiliations:** grid.417808.2 0000 0001 1393 1398 A.V. Zhirmunsky National Scientific Center of Marine Biology, Far Eastern Branch of the Russian Academy of Sciences (FEB RAS), 17 Palchevskogo Street, Vladivostok, Russian Federation 690041

**Keywords:** Biochemistry, Ecology

## Abstract

The cold-water gorgonian coral *Paragorgia arborea* is considered as a foundation species of deep-sea ecosystems in the northern Atlantic and Pacific oceans. To advance lipidomic studies of deep-sea corals, molecular species compositions of diacylglycerol ethers (DAGE), which are specific storage lipids of corals, and structural glycerophospholipids (GPL) including ethanolamine, choline, inositol and serine GPL (PE, PC, PI, and PS, respectively) were analyzed in *P. arborea* by HPLC and tandem mass spectrometry. In DAGE molecules, alkyl groups (16:0, 14:0, and 18:1), polyunsaturated fatty acids (PUFA), and monounsaturated FA are mainly substituted the glycerol moiety at position *sn*-1, *sn*-2, and *sn*-3, respectively. The ether form (1-*O*-alkyl-2-acyl) predominates in PE and PC, while PI is comprised of the 1,2-diacyl form. Both ether and diacyl forms were observed in PS. At position *sn*-2, C_20_ PUFA are mainly attached to PC, but C_24_ PUFA, soft coral chemotaxonomic markers, concentrate in PS, PI, and PE. A comparison of non-polar parts of molecules has shown that DAGE, ether PE, and ether PC can originate from one set of 1-*O*-alkyl-2-acyl-*sn*-glycerols. Ether PE may be converted to ether PS by the base-exchange reaction. A diacylglycerol unit generated from phosphatidic acid can be a precursor for diacyl PS, PC, and PI. Thus, a lipidomic approach has confirmed the difference in biosynthetic origins between ether and diacyl lipids of deep-sea gorgonians.

## Introduction

Lipidomic studies of organisms, cells, or tissues begin with a quantitative characterization of full profiles of certain lipid molecules commonly referred to as lipid molecular species^1^. The sustained efforts of numerous research groups resulted in the development of robust methods and mass spectrometry libraries for analyzing lipid molecular species related to human health and diseases^2^. These methods and libraries can be applied to most eukaryotic systems^3–5^ with, however, some restrictions in the case of marine invertebrates^6^, which additionally contain fatty acids and lipid classes with specific chemical structures^7,8^.

Marine invertebrates are a paraphyletic systematic group comprising more than 90% of all marine animal species, but data on their lipidomes are very limited to date^9^. These lipidomes have been partly described from some species of shrimp^10–12^, lobsters^13^, crabs^14,15^, jellyfish^16,17^, starfish^18^, sea urchins^18^, holothurians^18^, bivalves^19–25^, gastropods^26^, nudibranch molluscs^27,28^, sponges^29^, and hydrocorals^30,31^. Among coral polyps (the class Anthozoa), lipidome or its polar part has been analyzed in some species belonging to four orders: Scleractinia (reef-building corals)^32–34^, Alcyonacea (soft corals)^35–37^, Zoantharia^38^, and Actiniaria (sea anemones)^39–41^. No lipidome data have been obtained for members of other Anthozoa orders such as Gorgonacea, Pennatulacea, Antipatharia, and Ceriantharia.

Studies of lipid molecular species, in addition to lipid classes and fatty acids (FA) of total lipids, expand our knowledge on lipid biochemistry and ecology of corals. A lipidomic analysis of waxes showed that a bacterial community provides a greater contribution to storage lipids in asymbiotic coral species than in corals with symbiotic dinoflagellates^42^. The influence of environmental parameters and pollutions on cell physiology was assessed by analyzing the lipidome of reef-building corals^32–34,43^. Different patterns of distribution of polyunsaturated FA (PUFA) among structural lipid classes were demonstrated for different taxa of soft corals and hydrocorals from tropical and cold-water regions^30^. A lipidome analysis allowed simultaneous monitoring of lipid dynamics of both symbiotic dinoflagellates and host tissues during coral bleaching^44,45^. An in situ lipidome analysis of symbiotic dinoflagellates revealed possible mechanisms of heat stress tolerance in reef-building corals^46^. The accumulation of dietary very-long-chain FA (VLCFA) in nudibranch mollusks preying on soft corals was explained by comparing the lipidomes of soft corals and predators^28^.

In corals, a lipidomic comparison between different phospholipid classes has been applied for resolving biosynthetic relationships of these classes^30,47^. The major storage lipid classes of corals are wax esters, triacylglycerols, and specific ether lipids such as 1-*O*-alkyl-2,3-diacyl-*sn*-glycerols or diacylglycerol ethers (DAGE)^48^. The structural lipids classes of corals include ceramide aminoethylphosphonate (CAEP) and four glycerophospholipid (GPL) classes: ethanolamine, choline, inositol and serine GPL (abbreviated here as PE, PC, PI, and PS, respectively)^30^. Besides 1,2-diacyl GPL, corals contain also large amounts of ether phospholipids, which are divided into two types, plasmanyl (alkyl moiety at position *sn*-1, 1-*O*-alkyl-2-acyl GPL), and plasmenyl (alkenyl moiety with vinyl ether linkage at position *sn*-1, 1-*O*-(alk-1´-enyl)-2-acyl GPL)^30^. According to general biosynthetic pathways, 1-*O*-alkyl-2-acyl-*sn*-glycerol is converted into PE and PC by enzyme systems used to produce diacyl forms^49,50^. Presumably, 1-*O*-alkyl-2-acyl-*sn*-glycerols are the main source of DAGE in animal cells^49^. Hence, a comparison of non-polar parts of lipid molecules (alkyl/acyl composition) can confirm a hypothesis that DAGE and alkyl/acyl PE and PC in coral cells originated from a single precursor, 1-*O*-alkyl-2-acyl-*sn*-glycerol. Chemical structures of DAGE and triacylglycerols are similar and, therefore, DAGE may be a form of coral storage lipids. However, data on DAGE molecular species composition of corals still remains very limited^37,44^.

The cold-water gorgonian coral *Paragorgia arborea* (Paragorgiidae) mainly inhabits a depth range between 50 and 1300 m. This coral is widely distributed in the northern Atlantic and northern Pacific oceans, and is considered as a foundation species of deep-sea ecosystems^51^. The FA and lipid class compositions of *P. arborea* were described earlier^52,53^. In the present study, the profile of the lipid molecular species of phospholipid classes and DAGE was analyzed by a combination of high-performance liquid chromatography (HPLC) and tandem mass spectrometry (MS/MS). The lipidomics strategy was applied and individual molecular species were manually identified. A comparison of the lipidomes of the gorgonian coral *P. arborea* and other corals was carried out. Similarities of non-polar parts of molecules of each lipid class of *P. arborea* were tested to confirm possible biosynthetic relationships between the coral lipid classes.

## Results

### Fatty acid, alkylglycerol, and molecular species compositions of DAGE

Following the common way, we first determined the composition of acyl and alkyl groups of DAGE molecules in *P. arborea*. The major FA were 18:1n-9, 20:4n-6, 20:5n-3, and 16:0 (26.0, 18.3, 9.9, and 9.4% of total FA, respectively). Two VLCFA (24:5n-6 and 24:6n-3) were detected (Supplementary Table S1, Fig. S1, S2). Compounds with 16:0, 14:0, and 18:1 alkyl groups dominated total 1-*O*-alkyl-*sn*-glycerols (71.8, 10.3, and 9.3%, respectively) (Supplementary Table S1, Fig. S3, S4). The position of double bonds in the alkyl chains was not determined.

**Figure 1:**
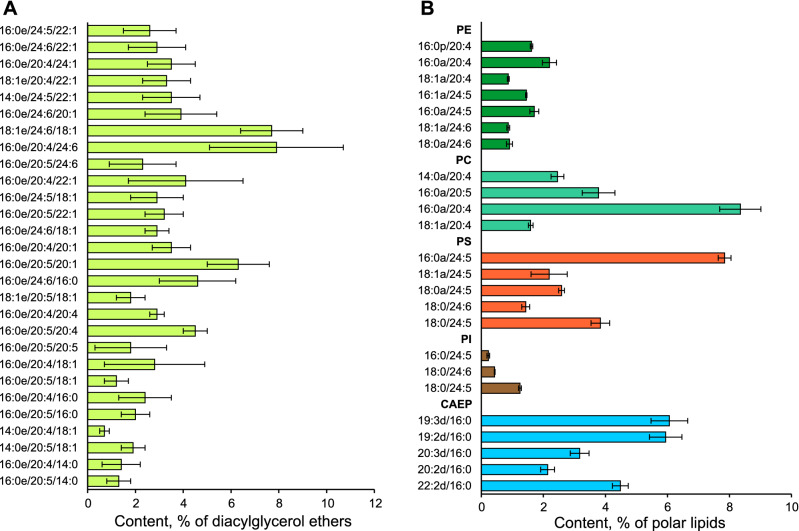
DAGE and polar lipid molecular species. Content of molecular species of (**A**) diacylglycerol ethers (% of total DAGE, mean ± SD, *n* = 6) and (**B**) polar lipid classes (% of total polar lipids, mean ± SD, *n* = 3) in the soft coral *Paragorgia arborea*. The polar lipids are comprised of ceramide aminoethylphosphonate (CAEP), ethanolamine, choline, inositol, and serine glycerophospholipids (PE, PC, PI, and PS, respectively). DAGE molecules are abbreviated as Xe/Y/Z, where Xe is alkyl group, Y and Z are acyl groups (number of carbon atoms : number of double bonds) at *sn*-1/*sn*-2/*sn*-3 positions of the glycerol ether backbone. CAEP molecules are abbreviated as Xd/Y; alkyl/acyl, alkenyl/acyl, and diacyl molecules of glycerophospholipids, as Xa/Y, Xp/Y, and X/Y, respectively. Xd, sphingoid base; Xa, alkyl group; Xp, alk-1-enyl group; X and Y, acyl groups.

The composition of DAGE molecular species is shown in Fig. 1A and Supplementary Table S2. The letter “e” after the first fatty acids in DAGE means 1-*O*-alkyl group. The distribution of alkyl and acyl groups at positions *sn*-1/*sn*-2/*sn*-3 of a glycerol ether backbone of each DAGE molecular species was determined (Supplementary Fig. S5). Three *O*-alkyl groups such as hexadecyl (16:0), tetradecyl (14:0), and octadecenyl (18:1) mainly attached to glycerol at position *sn*-1. The proportions of DAGE molecules with these major *O*-alkyl groups showed no significant (ANOVA, *p* > 0.01) differences from the proportions of the corresponding 1-*O*-alkyl-*sn*-glycerols obtained by hydrolysis of DAGE. Only polyunsaturated FA (PUFA) were found at position *sn*-2 of the glycerol backbone. At position *sn*-3, 60.2 ± 3.5% of DAGE molecules attached monounsaturated FA (MUFA), and 11.9 ± 3.6% of DAGE molecules attached saturated FA (SFA). VLCFA were found at both *sn*-2 and *sn*-3 of the glycerol backbone.

### Molecular species of polar lipids

The polar lipidome of *P. arborea* was described for the first time. Total polar lipids were composed of CAEP (29.4%), PE (15.4%), PC (28.1%), PS (24.2%), and PI (2.8%) (Supplementary Fig. S6). A total of 239 molecular species of polar lipids were identified (Supplementary Table S3, Supplementary Fig. S7). The letter “a” after the first fatty acids in GPL means 1-*O*-alkyl group (plasmanyl form), the letter “p”—plasmenyl form, 1-*O*-alk-1´-enyl group. The contents of 22 major molecular species of five polar lipid classes (more than 5% of each lipid class) are shown in Fig. 1B. These major molecular species made up 67% of total polar lipids. CAEP molecular species differed in sphingoid bases, but only palmitic acid (16:0) was detected as *N*-acyl groups. The letter “d” means a sphingoid base in the names of CAEP molecules. The sphingoid bases had a 2-amino-1,3-dihydroxy long-chain core structure with 18–22 carbon atoms and 1–4 double bonds. Both ether and diacyl forms of GPL were identified. The ether form dominated PE, PC, and PS. The content of the ether molecular species in PE, PC, PS, and PI was 99.3, 85.4, 64.1, and 2.0%, respectively. The noticeable level of the alkenylacyl (plasmalogen) form was found only in PE and PS (29.7 and 5.9% of lipid class, respectively).

Saturated and monounsaturated C_16_ and C_18_ alkyl/acyl groups were most abundant at position *sn*-1 in GPL molecules. Clear differences in the PUFA composition between GPL classes were observed. At position *sn*-2, C_20_ PUFA such as 20:4n-6 and 20:5n-3 dominated acyl groups in PC molecular species, while C_24_ VLCFA dominated acyl groups in PS and PI. Approximately half of PE molecules contained C_20_ PUFA at position *sn*-2, but 47% of PE molecules were esterified by C_24_ VLCFA such as 24:5n-6 and 24:6n-3.

### Comparison of non-polar parts of GPL and DAGE

**Figure 2:**
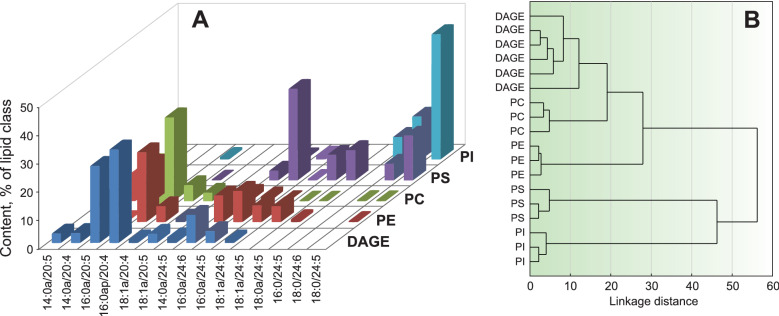
Comparison of non-polar parts of GPL and DAGE. (**A**) Profiles (% of lipid class) of 1-*O*-alkyl-2-acyl parts of glycerophospholipid (GPL) and diacylglycerol ether (DAGE) molecular species in the soft coral *Paragorgia arborea*. (**B**) A dendrogram of cluster analysis of 6 samples of DAGE and 12 samples of four GPL classes by their 1-*O*-alkyl-2-acyl part composition. Ethanolamine, choline, inositol and serine GPL are abbreviated as PE, PC, PI, and PS, respectively; alkyl/acyl and diacyl forms, as Xa/Y and X/Y; Xa, alkyl group; X and Y, acyl groups (number of carbon atoms: number of double bonds). 16:0ap/20:4 is the sum of plasmanyl and plasmenyl forms.

On the basis of the polar lipidome and the DAGE molecular species composition of *P. arborea*, the major molecular species of GPL and DAGE were compared according to the composition of alkyl and acyl groups at positions *sn*-1/*sn*-2 (Fig. 2A, Supplementary Table S4). A cluster analysis (Fig. 2B) revealed a similarity in the *sn*-1/*sn*-2 composition between DAGE, PC and PE. The molecular species 16:0a/20:4 (*sn*-1/*sn*-2) was most abundant in these three lipid classes. The dendrogram also shows that the group of DAGE, PC, and PE differed from PS and PI that contained high proportions of VLCFA at position *sn*-2 (Fig. 2A). The same diacyl forms (18:0/24:5 and 18:0/24:6) were observed in both PI and PS molecular species. Due to the trace amounts of PI ether forms, we do not consider the biosynthetic relationship between DAGE and PI below.

## Discussion

In addition to triacylglycerols, the common storage lipid class, corals contain diacylglycerol ethers (DAGE). Up to 50% of coral total lipids may be comprised of DAGE^48^. These two storage lipid classes have similar chemical structures but different biosynthetic pathways^54,55^. Coral colonies exposed to thermal stress demonstrate different dynamics of the triacylglycerol and DAGE catabolism^44^. The molecular species profiles of triacylglycerols and DAGE were earlier described from two alcyonarian species of the genus *Sinularia*^37,44^, the reef-building coral *Acropora cerealis*^45^, and the zoanthid *Palithoa tuberculosa*^38^. In these coral polyps, the PUFA acyl groups were mainly attached at positions *sn*-1(3) in triacylglycerols but at position *sn*-2 in DAGE molecules. This observation thereby confirms the different biosynthetic origins of these lipid classes in corals. A comparison of coral polyps showed order-specific differences in the FA composition at position *sn*-2 between DAGE profiles. SFA were mainly attached to the glycerol moiety in DAGE of tropical alcyonarians^37,45^, whereas PUFA dominated acyl groups at position *sn*-2 of DAGE in the tropical zoanthid *P. tuberculosa* and the deep-sea gorgonian *P. arborea*. Most of these PUFA are represented by long-chain acids (20:4n-6 and 20:5n-3). Furthermore, the markers of symbiotic zooxanthellae (18:3n-6, 18:4n-3, and C_16_ PUFA) were presented in DAGE of the zoanthid, while the chemotaxonomic markers of octocorals (24:5n-6 and 24:6n-3) were found in DAGE of *P. arborea*. Thus, medium-chain PUFA, which can be transferred from photosynthetic symbionts^56,57^ or possibly synthesized in the coral host tissues^58^, and polyunsaturated VLCFA, which are mainly concentrated in structural lipids, primarily PS^30^, can be incorporated at position *sn*-2 of DAGE molecules in coral polyps.

The esterification of the glycerol backbone of DAGE at positions *sn*-2 and *sn*-3 are catalyzed by different acyltransferases^54^. In brief, DAGE molecular species of the deep-sea coral *P. arborea* contain polyunsaturated acyl groups at position *sn*-2 and monounsaturated acyl groups at position *sn*-3. The same distribution of acyl groups between the *sn*-2 and *sn*-3 positions depending on the unsaturation degree was earlier observed in DAGE isolated from digestive glands of the deep-sea squid *Berryteuthis magister*^59^. Typically, lower water temperatures result in an increase in the lipid unsaturation level.

The distribution of PUFA among structural lipid classes in *P. arborea* was typical for the soft corals. It was found that C_20_ PUFA mainly located in PE and PC, while VLCFA concentrated in PS and, partly, in PI^30,35^. In contrast to PS of tropical alcyonarians, which contain only an ether form, both diacyl and ether forms of PS were observed in deep-sea *P. arborea*, similarly to the cold-water soft corals *Gersemia rubiformis* and *G. fruticosa*^30,36,47^. We suppose that the presence of diacyl PS is necessary to maintain the membrane fluidity of coral cells under lower water temperatures.

The high level of PE with VLCFA (46.6% of total PE) in *P. arborea* distinguishes this species from other corals studied. The PC molecular species with VLCFA were also found in *P. arborea*. In our earlier studies, PE and PC with VLCFA were not detected in corals^60^. Subsequently, PE 18:0ap/24:5 (the sum of plasmanyl and plasmenyl forms) was found in the tropical alcyonarians *Capnella* sp., *Sinularia siaesensis*, and *S. heterospiculata* (2.1, 5.9, and 10.8% of total PE, respectively)^35,37^. Thus, C_24_ PUFA can be incorporated in all structural lipid classes (except for CAEP) of soft corals. To recognize the high level of PE with VLCFA as a chemotaxonomic trait of the genus *Paragorgia*, further analyses of lipidomes of other tropical and cold-water gorgonian species are required.

According to the general views on ether lipid biosynthesis^54^, 1-*O*-alkyl-2-acyl-*sn*-glycerol can be esterified with a long-chain acyl-CoA ester, yielding a neutral 1-*O*-alkyl-2,3-diacyl-*sn*-glycerol (DAGE). The same alkylacylglycerol can be converted into 1-*O*-alkyl-2-acyl-*sn*-glycero-3-phosphoethanolamine (plasmanyl PE) or 1-*O*-alkyl-2-acyl-*sn*-glycero-3-phosphocholine (plasmanyl PC). A conversion of PE into PC by repeated methylation is also possible. The high similarity in the composition of 1-*O*-alkyl-2-acyl parts between the molecules of DAGE, plasmanyl PE, and plasmanyl PC confirms that both storage and structural ether lipid classes in the soft coral *P. arborea* have the same biosynthetic origin.

**Figure 3:**
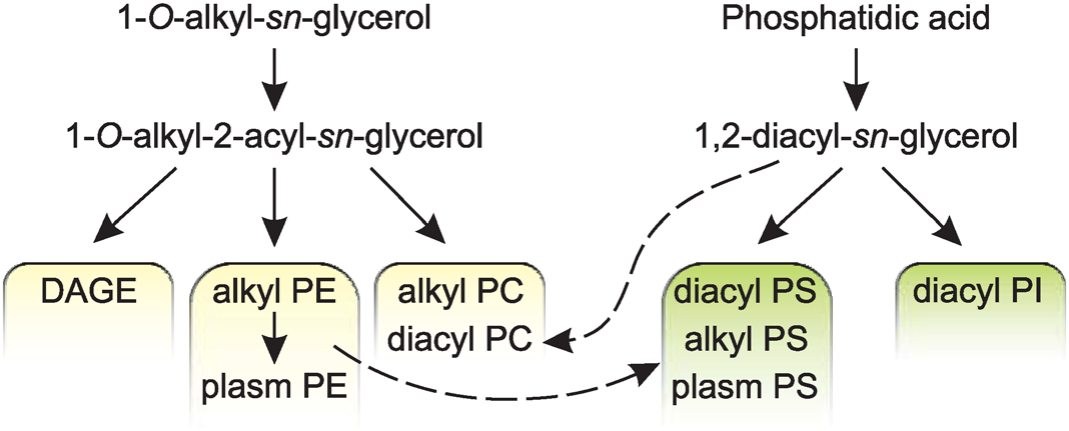
Biosynthesis of GPL and DAGE. Diagram of possible biosynthetic relationships between major classes of structural glycerophospholipids (GPL) and storage diacylglycerol ether (DAGE) in the soft coral *Paragorgia arborea*, as inferred from comparison of the compositions of the non-polar parts of their molecular species. Ethanolamine, choline, inositol and serine GPL are abbreviated as PE, PC, PI, and PS, respectively. Alkyl, 1-*O*-alkyl-2-acyl; diacyl, 1,2-diacyl; plasm, 1-*O*-(alk-1′-enyl)-2-acyl.

The plasmanyl PE (alkyl PE) can further be transformed into their corresponding plasmenyl form, 1-*O*-(alk-1′-enyl)-2-acyl-*sn*-glycero-3-phosphoethanolamine (plasm PE)^49^. In *P. arborea*, this transformation is confirmed by the high level of plasm PE. No enzymes converting plasmanyl PS (alkyl PS) into plasmenyl PS (plasm PS) are known to date, but PS can be synthesized by the base-exchange reaction between PE and serine^50^. We suppose that this reaction can lead to the conversion of a part of ether PE to ether PS in the soft coral *P. arborea*. It should be noted that this base-exchange reaction mainly involves the molecular species of PE with VLCFA at position *sn*-2. Possible pathways of synthesis of ether lipids in *P. arborea* are summarized in Fig. 3.

The synthesis of mammalian diacyl GPL requires either 1,2-diacylglycerol or CDP-diacylglycerol generated from phosphatidic acid^50^. A diacylglycerol unit can be a precursor in the synthesis of diacyl PI in the soft coral *P. arborea*. The same synthetic way or the base-exchange reaction between diacyl PI and serine may lead to the formation of diacyl PS in *P. arborea*. Similarly to ether PS, diacyl PS molecular species are esterified by VLCFA at position *sn*-2. Recently, a biosynthetic relationship between PS and PI in hydrocoral species has been reported on the basis of lipidomic data^30^. The reaction between diacylglycerol and CDP-choline may produce diacyl PC. Possible pathways of synthesis of diacyl GPL in *P. arborea* are shown in Fig. 3. Thus, a lipidomic approach has shown the biosynthetic origins of ether and diacyl lipids, confirmed the general pathways of lipid biosynthesis, and revealed relationships between DAGE and GPL, as well as among GPL classes, in gorgonian corals.

## Methods

### Chemicals

The use of chemicals was described previously^28,47^. All solvents were of HPLC or LCMS grade. Polar lipid standards (16:0–20:4 PC, 16:0–20:4 PE, 16:0–20:4 PS, 18:0–20:4 PI, C18(Plasm)-20:4 PC, C18(Plasm)-20:4 PE, and C16-18:1 PC) were purchased from Avanti Polar Lipid Co. (Alabaster, USA). Neutral lipid standards (1-*O*-hexadecyl-2,3-dihexadecanoyl-*rac*-glycerol and glycerol trioleate) and a mixture of PUFA methyl esters No. 3 from menhaden oil were purchased from Sigma-Aldrich Co. (St. Louis, USA). Silica gel 60 (230–400 mesh) for column chromatography were obtained from Merck (Darmstadt, Germany). Silica gel TLC plates (PTLC-AF-V) with a silica sol binder on aluminum foil were provided by Sorbfil (Krasnodar, Russian Federation).

### Collection of specimens

Specimens of the gorgonian coral *Paragorgia arborea* (Linnaeus, 1758) were collected with a dredge off Urup Island (Kuril Islands, 45° 49′ N, 149°36′ E) at a depth of 170–200 m in July 2019. Six specimens were used for the DAGE molecular species analysis; three specimens were used for the FA, alkylglycerol, CAEP, and phospholipid molecular species analysis.

### Lipid extraction

Total lipids were extracted from fresh coral samples immediately aboard the research vessel. The extraction technique of Folch et al.^61^ was modified according to Imbs and Chernyshev^47^. The samples were homogenized in a chloroform:methanol (1:2, *by vol.*) mixture (30 mL per 10 g wet weight). The obtained homogenate was filtered, and the residue was repeatedly extracted (6 h, 4 °C) in a chloroform:methanol (2:1, *by vol.*) mixture (2 × 30 mL). The extracts were then mixed and separated into layers by adding 35 mL of water and 30 mL of chloroform. The lower layer was separated and evaporated. Total lipids were dissolved in chloroform and stored at − 80 °C.

### Isolation of DAGE

DAGE were isolated from the total lipids by a low-pressure column chromatography on silica gel. The DAGE were eluted with hexane–diethyl ether (95:5, *by vol.*) after hydrocarbons and triacylglycerols. The composition of chromatographic fractions (triacylglycerols, R_f_ 0.58; DAGE, R_f_ 0.44) was controlled by TLC developed in hexane:diethyl ether (90:10, *by vol.*). The fractions with pure DAGE were combined, evaporated under vacuum, dissolved in chloroform, and stored at − 80 °C.

### Lipid class analysis

Polar lipid class analysis was performed on a Shimadzu liquid chromatograph (Kyoto, Japan) equipped with two LC-20AD pump units, a high-pressure gradient forming module, a CTO-20A column oven, a SIL-30AC auto sampler, a CBM-20A communications bus module, a DGU-20A5R degasser, and a low-temperature evaporating light scattering detector ELSD LT II (Shimadzu, Japan) (the temperature of evaporating tube was 65 °C, the pressure of spraying gas (N_2_) was 300 kPa). A column Develosil 100–5 (150 mm × 2 mm ID, 5 μm particle size, Nomura Chemical, Seto, Japan) was operated in HILIC mode at temperature 40 °C. A binary gradient of mobile phase A (acetonitrile containing 50 mM formic acid) and mobile phase B (acetonitrile:water (1:1, *by vol.*) containing 100 mM formic acid and 40 mM ammonia) were used. The mixer volume was 40 µL. The elution program was set as a linear gradient between time points: 0 min (5% B, total flow rate 0.2 mL min^−1^), 1.6 min (22% B, total flow rate was reduced from 0.2 to 0.1 mL min^−1^), 7 min (38% B), 13 min (46% B), 14 min (60% B), 18 min (60% B), 18.01 min (100% B), 21 min (total flow rate was increased from 0.1 to 0.3 mL min^−1^), 22 min (100% B), 22.01 min (5% B), and 23.5 min (total flow rate was reduced from 0.3 to 0.2 mL min^−1^). A solution (1 µL) of total lipids in chloroform (1 mg mL^−1^) was injected.

### Analyses of fatty acid, alkylglycerol, and molecular species compositions of DAGE

Fatty acid methyl esters (FAME) were obtained by acidic methanolysis of pure DAGE as described previously^28^. 4,4-Dimethyloxazoline (DMOX) derivatives of FA were prepared according to Svetashev^62^. A GC analysis of FAME and GC−MS analysis of FAME and DMOX derivatives were conducted according to Imbs et al.^28^ The methods described by Rybin et al.^59^ were used for obtaining 1-*O*-alkyl-glycerols by alkaline hydrolysis of DAGE, preparing trimethylsilyl derivatives of alkylglycerols followed by GC−MS analysis, and analyzing DAGE molecular species by HPLC on a high-resolution tandem ion-trap/time-of-flight mass spectrometer. All conditions of analyses by GC, GC–MS, and HPLC–HRMS/MS are described in detail in the Supplementary materials.

### Analysis of polar lipid molecular species

Lipids were separated on a Shimadzu liquid chromatograph (Kyoto, Japan), equipped with four LC-30AD pump units, a high-pressure gradient forming module, a CTO-20AC column oven, a SIL-30AC auto sampler, a CBM-20A communications bus module, DGU-20A3R and DGU-20A5R degassers, and an Ascentis Express C18 column (150 mm × 2.1 mm ID, 5 μm particle size, Supelco, Bellefonte, USA) operated at 70 °C. Four mobile phases were used: A, methanol; B, 2-propanol; C, water containing 2 M formic acid and 1.8 M ammonia; D, water. Channels A, B, and D were connected to a mixer (40 µL) through cartridge (10 mm × 2 mm ID) with SCX-1001 cationite (Yanaco, Japan), and channel C was connected directly to the mixer. Eluent was pumped at a constant flow of 0.2 mL min^−1^ with a stepwise gradient (% of A:B:C:D, *by vol.*): 0 min (33.75:41.25:2.5:22.5), 5 min (28.5:46.5:2.5:22.5), 15 min (24.75:50.25:2.5:22.5), 20 min (11.25:63.75:2.5:22.5), 22 min (0:75:2.5:22.5), 30 min (0:82.5:2.5:15), 35 min (0:100:0:0), and 45 min (33.75:41.25:2.5:22.5). The eluent outlet was coupled with the mass-spectrometer.

Quantitative analyses of molecular species and their identification using fragmentation pattern were performed on a Shimadzu LCMS-8060 triple-quadrupole mass spectrometer (Kyoto, Japan) operated in electrospray ionization (ESI) conditions. The temperature of the interface, heat block, and desolvation line was 300, 400, and 250 °C, respectively. The drying gas (N_2_) flow was 10 L min^−1^. The nebulizer gas (N_2_) flow rate was 3 L min^−1^. The heating gas (zero air) flow rate was 10 L min^−1^. The negative ion mode was applied for analyzing PI, and the positive mode was applied for analyzing others polar lipid classes. CAEP, PE, and PS were detected by scanning for a neutral loss of 125, 141, and 185 Da, respectively, for their precursor ions^63–65^. Detection of specific fragment ions such as *m/z* 184 for PC and *m/z* 241 for PI was performed^63,64^. Collision energy (CE) in characteristic fragmentation reactions of CAEP, PE, PC, PS, and PI was set at − 23 V, − 24 V, − 33 V, − 23 V, and 44 V, respectively; scan speed was 3000 amu s^−1^. Fragmentation parameters (product ion scan range, CE) were set for each lipid class as follows: CAEP, *m/z* 165–500, –35 V; PE, *m/z* 256–700, − 26 V; PC, *m/z* 256–700, − 30 V; PS, *m/z* 228–860, –24 V; PI, *m/z* 171–425, 50 V. Scan speed was 15,000 amu sec^−1^.

To determine exact molecular masses of polar lipids, we carried out high-resolution tandem ion trap–time of flight mass spectrometry on a Shimadzu LCMS-IT-TOF instrument (Kyoto, Japan) operated in the positive and negative ion modes during each analysis in ESI conditions. The ion source temperature was 200 °C; the range of detection was *m/z* 600–1100; the potential in the ion source was − 3.5 for the negative mode and 4.5 kV for the positive mode. The drying gas (N_2_) pressure was 100 kPa. The nebulizer gas (N_2_) flow rate was 1.5 L min^−1^. Precise molecular masses were used to calculate the brutto-formulas and distinguish diacyl lipids and ether lipids. The chemical structure of the polar lipid molecular species was identified as described earlier (Supplementary Fig. S7)^65,66^.

To confirm the presence of molecular species with the plasmalogen structure, the lipidomic profiles of samples were compared before and after mild acid hydrolysis. In brief, about 50 µg of the lipid sample was evaporated to dryness under argon stream in a glass vial (1 mL). The vial was placed bottom up on the drop of concentrated HCl for 3 min. Then HCl was blown off with argon stream, lipids dissolved in 50 µL of chloroform and analysed again by LC–MS. The disappearance of peaks of certain lipid molecular species on the chromatogram confirms the plasmalogen structure of these molecules.

### Statistical analysis

The analysis approach was recently described^28^. In brief, significance of differences between mean contents of DAGE and alkylglycerols was tested by one-way analysis of variance (ANOVA). Raw data were used following evaluation of the homogeneity of variances (Levene’s test) and the normality of data distribution (Shapiro–Wilk test). For the cluster analysis, the unweighted pair-group method with arithmetic mean and the Euclidean distance as dissimilarity metric were applied to the percentage content of molecular species of DAGE and four GPL classes according to the structure of their non-polar parts. All statistical analyses were performed using STATISTICA 5.1 (StatSoft, Inc., USA). A statistical probability of *p* < 0.01 was considered significant. Values are represented as mean ± standard deviation.

## Supplementary Information

Supplementary Information.
