# Supplementary material for: Lipidomic profiling reveals biosynthetic relationships between phospholipids and diacylglycerol ethers in the deep-sea soft coral Paragorgia arborea
Source: Sci Rep. 2021 Oct 28;11:21285. doi: 10.1038/s41598-021-00876-5 (PMC8553863; doi:10.1038/s41598-021-00876-5)
Supplement: Supplementary file 1 — Supplementary Information. [file 41598_2021_876_MOESM1_ESM.pdf]

Supplementary information

Lipidomic profiling reveals biosynthetic relationships between phospholipids and diacylglycerol ethers in the deep-sea soft coral *Paragorgia arborea*

Andrey B. Imbs & Peter V. Velansky

Method Section

Fatty acid analysis. Fatty acid methyl esters (FAME) were obtained by a treatment of DAGE with 2% H2SO4/MeOH at 80°C for 2 h in a screw-caped vial under argon, extracted with hexane and purified by preparative TLC developed in benzene. 4,4-Dimethyloxazoline (DMOX) derivatives of FA were prepared according to Svetashev (2011). A gas chromatography analysis of FAME was conducted with a GC-2010 chromatograph (Shimadzu, Kyoto, Japan) with a flame ionization detector (Imbs & Grigorchuk, 2019). An Equity-5 (Supelco, Bellefonte, USA) capillary column (30 m × 0.25 mm i.d., film thickness 25 μm) was held for 2 min at 170°C, then heated with a 2°C min^{-1} ramp to 240°C that was held for 5 min. The injector (250°C) and detector (260°C) temperatures were constant. Helium was used as the carrier gas at a linear velocity of 30 cm s^{-1}. Identification of FAs was confirmed by GC–MS of their FAME and DMOX derivatives using a GCMS-2010 Ultra instrument (Shimadzu, Kyoto, Japan) (electron impact at 70 eV) and a MDN-5ms (Supelco, Bellefonte, USA) capillary column (30 m × 0.25 mm i.d., film thickness 25 μm). Carrier gas was He at 30 cm s^{-1}. The GC–MS analysis of FAME was performed at 160°C with a 2°C min^{-1} ramp to 240°C that was held for 20 min. The injector and detector temperatures were 250°C. GC–MS of DMOX derivatives was performed at 210°C with a 3°C min^{-1} ramp to 270°C that was held for 40 min. The injector and detector temperatures were 270°C. Spectra were compared with the NIST library and mass spectra archive.

Alkylglycerol analysis. 1-*O*-alkyl-glycerols were obtained by treatment of DAGE with 1N NaOH/MeOH according to Hanuš et al. (2009). The alkylglycerols were converted to their trimethylsilyl derivatives by 200 μL of *N,O*-bis(trimethylsilyl)acetamide and 100 μL of chlorotrimethylsilane; the reaction was carried out at 70°C for 20 min in a screw-caped vial under argon. After addition of 200 μL of diethyl ether, 1 μL of the silylated fraction was injected into a Shimadzu GCMS-QP5050A instruments (Kyoto, Japan) with a SLB-5ms capillary column (30 m × 0.25 mm i.d., film thickness 25 μm; Supelco, Bellefonte, USA). The GC oven program had an initial temperature of 200°C, a 2°C min^{-1} run to 290°C, and a final hold at 290°C for 20 min. The injector temperature was 270°C (split 1:14), the interface temperature was 240°C, and the carrier gas (helium) flow rate was 28.2 cm s^{-1}. The scan range was from 50 to 650 m/z, the solvent delay was 3 min.

Analysis of DAGE molecular species. Analysis was performed on a Shimadzu LCMS-IT-TOF (Kyoto, Japan) system equipped with a LC-20A Prominence chromatograph and high-resolution tandem ion-trap/time-of-flight mass spectrometer according to Rybin et al. (2017). Separation of DAGE molecular species were performed on an Ascentis C18 column (100 mm × 2.1 mm i.d.; 3 μm particle size) at 40°C. The solvent flow rate was 0.2 mL min^{-1}. HPLC was carried out according to Mu et al. (2000) with a modified eluent composition. Solvent A: acetonitrile; solvent B: propan-2-ol–hexane (2:1, by vol.). Water (0.1% by volume) was added to all solvents. Compounds were eluted isocratically with a solvent A–solvent B mixture (90:10, by vol.) for 5 min, followed by a linear gradient to a solvent A–solvent B mixture (65:35, by vol.) for 40 min, and kept in solvent B (100%) for 5 min before returning to the initial solvent system. The range of detection was *m/z* 400–1200 (APCI, positive and negative ion detection). The potential in the ion

source was –4.5 kV. The drying gas (N_2) pressure was 25 kPa. The nebulizer gas (N_2) flow rate was 2 L min^{-1}. The interface temperature was 300°C. The collision gas (Ar) concentration was 50%.

The HRMS spectra of each molecular species of DAGE contained positive quasi-molecular ion $[M+H]^+$ with fragment ions $[M–R'CO_2]^+$, $[M–R''CO_2]^+$, and $[M–OR''']^+$, as well as negative cluster ion $[M+2H_2O–H]^-$ without fragmentation. The negative ions provided the precise values of monoisotopic molecular masses of all the components. The regiospecific positions of FAs and alkyl groups in DAGE molecules were determined according to Hartvigsen et al. (2001). In brief, the positive fragment ion with the highest intensity was assigned to the fragment of DAGE after elimination of a FA molecule from the *sn*-2 position. The positive fragment ion with a lower intensity (less than 50%) was assigned to the fragment of DAGE after elimination of a FA molecule from the *sn*-3 position. The positive fragment ion with the lowest intensity (10% and less) appeared after elimination of an alcohol molecule from the *sn*-1 position of the quasi-molecular ion.

References of the Method Section

Hanuš, L. O., Levitsky, D. O., Shkrob, I. & Dembitsky, V. A. Plasmalogens, fatty acids and alkyl glyceryl ethers of marine and freshwater clams and mussels. *Food Chem.* **16**, 491–498 (2009).
Hartvigsen, K., Ravandi, A., Bukhave, K., Holmer, G. & Kuksis, A. Regiospecific analysis of neutral ether lipids by liquid chromatography/electrospray ionization/single quadrupole mass spectrometry: validation with synthetic compounds. *J. Mass Spectrom.* **36**, 1116–1124 (2001).
Imbs, A. B. & Grigorchuk, V. P. Lipidomic study of the influence of dietary fatty acids on structural lipids of cold-water nudibranch molluscs. *Sci. Rep.* **9**, 20013, doi:10.1038/s41598-019-56746-8 (2019).
Mu, H., Kalo, P., Xu, X. & Høy, C.-E. Chromatographic methods in the monitoring of lipase-catalyzed interesterification. *Eur. J. Lipid Sci. Tech.* **102**, 202–211 (2000).
Rybin, V. G., Imbs, A. B., Demidkova, D. A. & Ermolenko, E. V. Identification of molecular species of monoalkyldiacylglycerol from the squid *Berryteuthis magister* using liquid chromatography–APCI high-resolution mass spectrometry. *Chem. Phys. Lipids* **202**, 55–61, doi: 10.1016/j.chemphyslip.2016.11.008 (2017).
Svetashev, V. I. Mild method for preparation of 4,4-dimethyloxazoline derivatives of polyunsaturated fatty acids for GC–MS. *Lipids* **46**, 463–467, https://doi.org/10.1007/s11745-011-3550-4 (2011).

Supplementary Table S1. Fatty acid and alkyl group composition (% of total, mean ± SD, n = 3) of diacylglycerol ethers (DAGE) in the soft coral *Paragorgia arborea*. Fatty acid methyl esters were obtained by acidic methanolysis of DAGE, 1-*O*-alkyl-*sn*-glycerols were obtained by alkaline hydrolysis of DAGE.

Fatty acid	Content, %	Alkyl group	Content, %
14:0	3.2 ± 0.5	14:0	10.3 ± 2.5
16:0	9.4 ± 1.2	15:0	1.5 ± 0.3
16:1n-7	3.1 ± 0.3	16:0	71.8 ± 8.4
18:0	2.5 ± 0.9	16:1	2.0 ± 0.2
18:1n-9	26.0 ± 5.3	17:0	1.0 ± 0.5
20:1n-11	2.3 ± 0.3	17:1	0.1 ± 0.1
20:1n-9	1.6 ± 0.2	18:0	2.8 ± 2.2
20:1n-7	2.8 ± 0.8	18:1	9.3 ± 3.1
20:4n-6	18.3 ± 5.3	18:2	0.2 ± 0.2
20:5n-3	9.9 ± 0.5	20:0	0.1 ± 0.1
22:1n-9	2.0 ± 0.2	20:1	0.6 ± 0.2
22:1n-7	2.9 ± 0.7	Other	0.4 ± 0.1
22:6n-3	2.6 ± 0.6		
24:5n-6	2.1 ± 0.8		
24:6n-3	2.9 ± 1.3		
Other	8.6 ± 1.7		

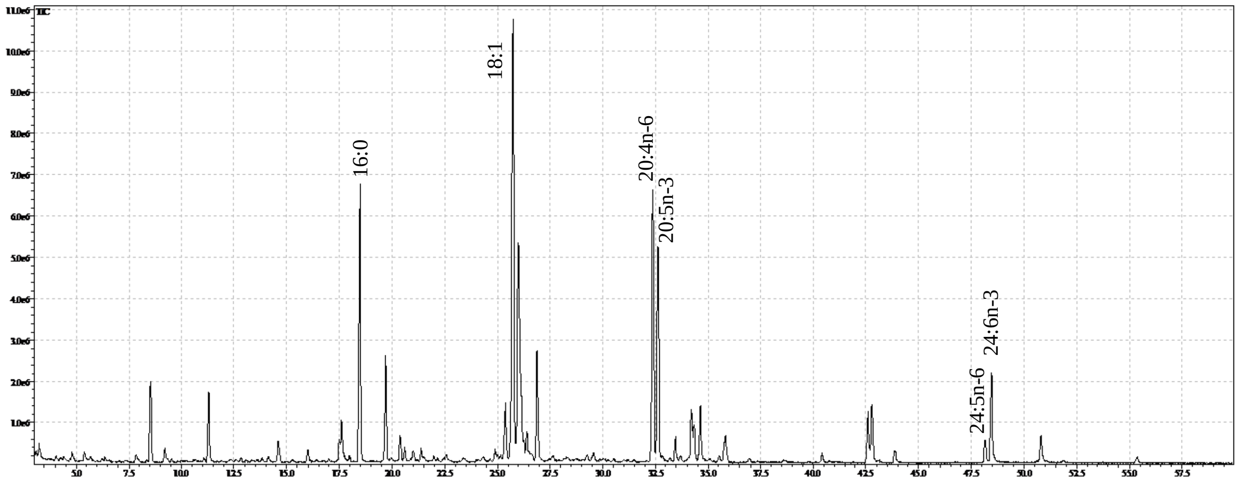

Supplementary Figure S1. A GC–MS chromatogram of fatty acid methyl esters (FAME) obtained by hydrolysis of diacylglycerol ethers (DAGE) of the soft coral *Paragorgia arborea*.

FAME 20:4n-6

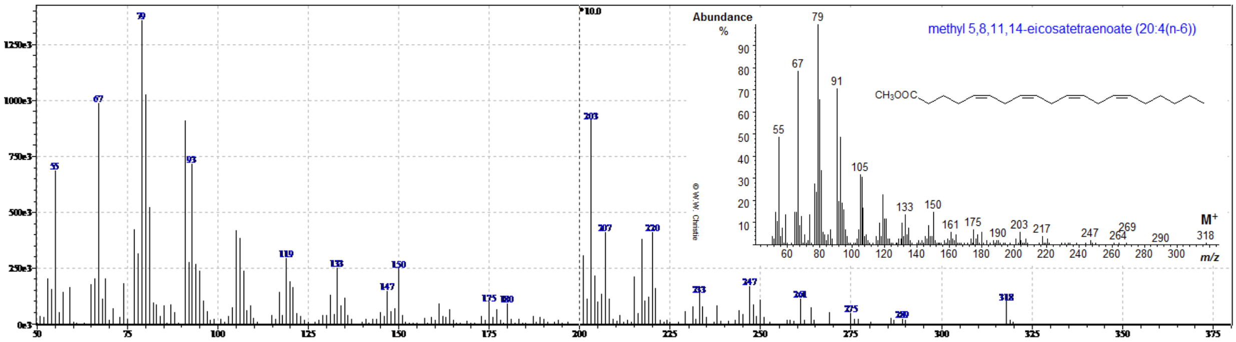

FAME 20:5n-3

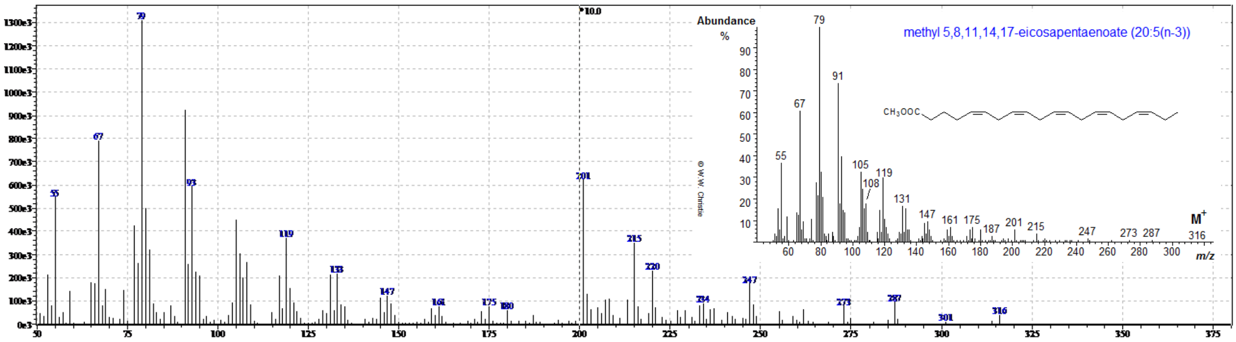

FAME 24:5n-6

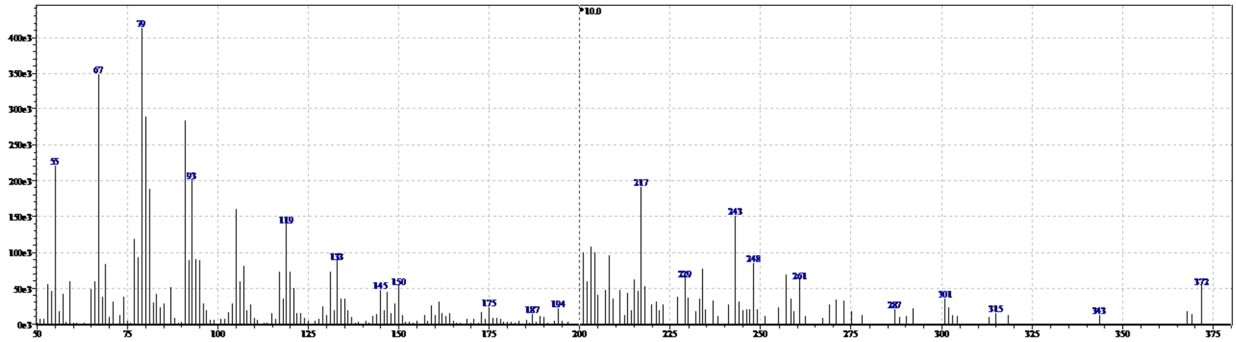

FAME 24:6n-3

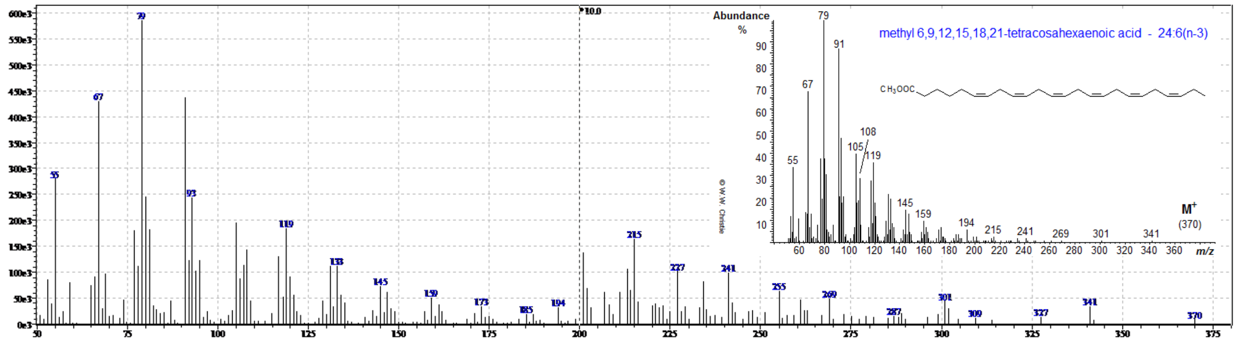

DMOX 20:4n-6

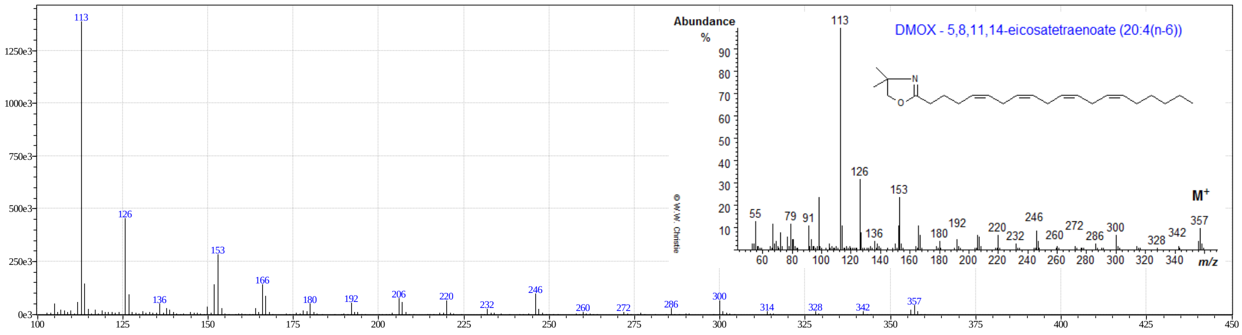

DMOX 20:5n-3

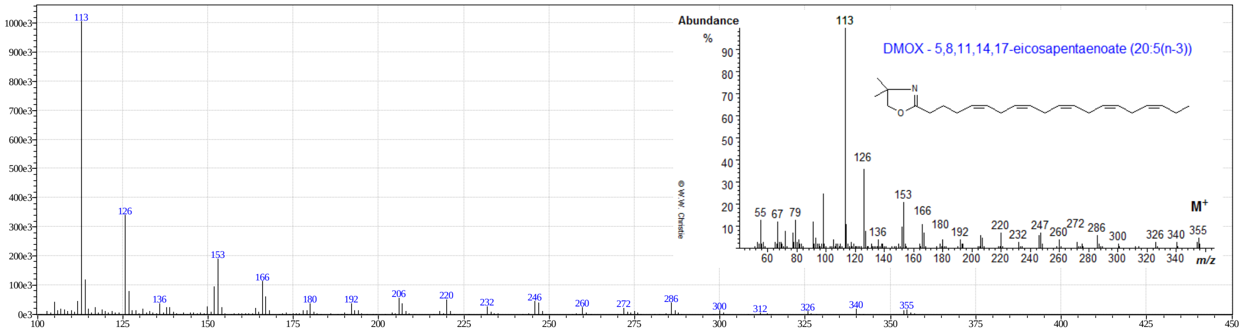

DMOX 24:5n-6

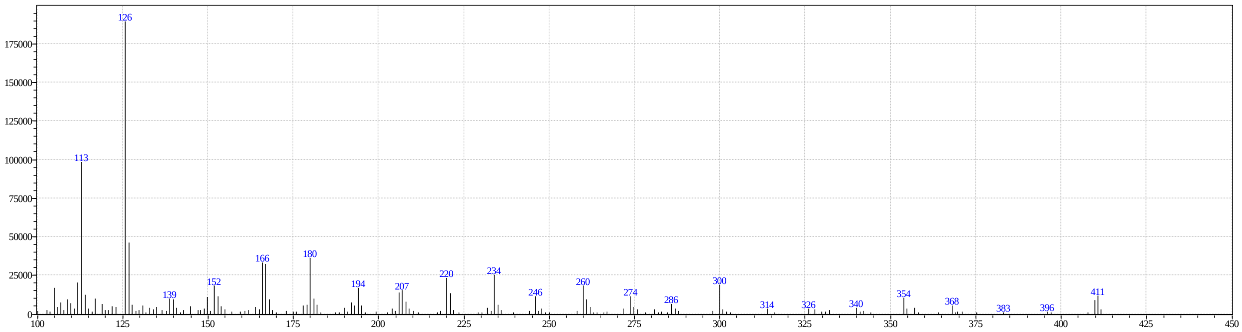

DMOX 24:6n-3

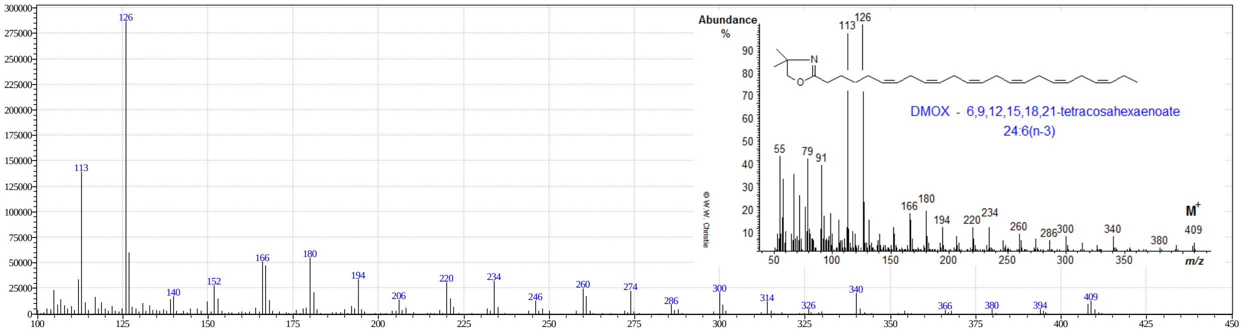

Supplementary Figure S2. Mass spectra of major polyunsaturated fatty acid methyl esters (FAME) and their 4,4-dimethyloxazoline derivatives (DMOX) obtained from diacylglycerol ethers (DAGE) of the soft coral *Paragorgia arborea*. For reference, the correspondence spectrum from the Archive of mass spectra were placed in the upper right corner of each spectrum obtained. (Christie, W.W. The LipidWeb. Archive of mass spectra. (2021) https://www.lipidmaps.org/resources/lipidweb/index.php?page=ms/methylesters.htm; https://www.lipidmaps.org/resources/lipidweb/index.php?page=ms/dmox.htm).

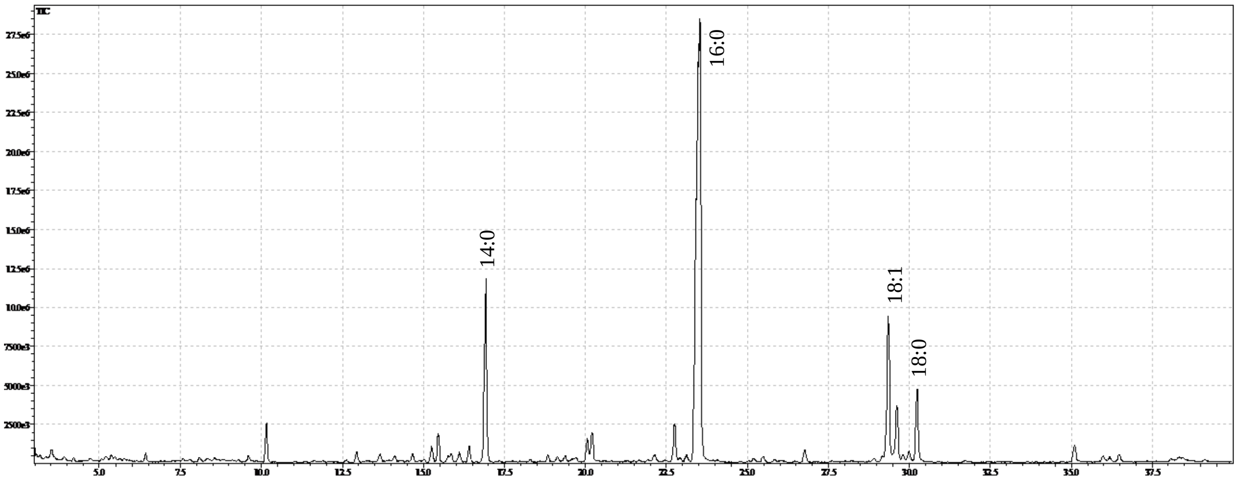

Supplementary Figure S3. A GC–MS chromatogram of trimethylsilyl (TMS) derivatives of 1-*O*-alkyl-*sn*-glycerols obtained by alkaline hydrolysis of DAGE of the soft coral *Paragorgia arborea*. Names of alkyl groups are indicated.

1-*O*-hexadecyl-2,3-*O*-di(trimethylsilyl)-*sn*-glycerol

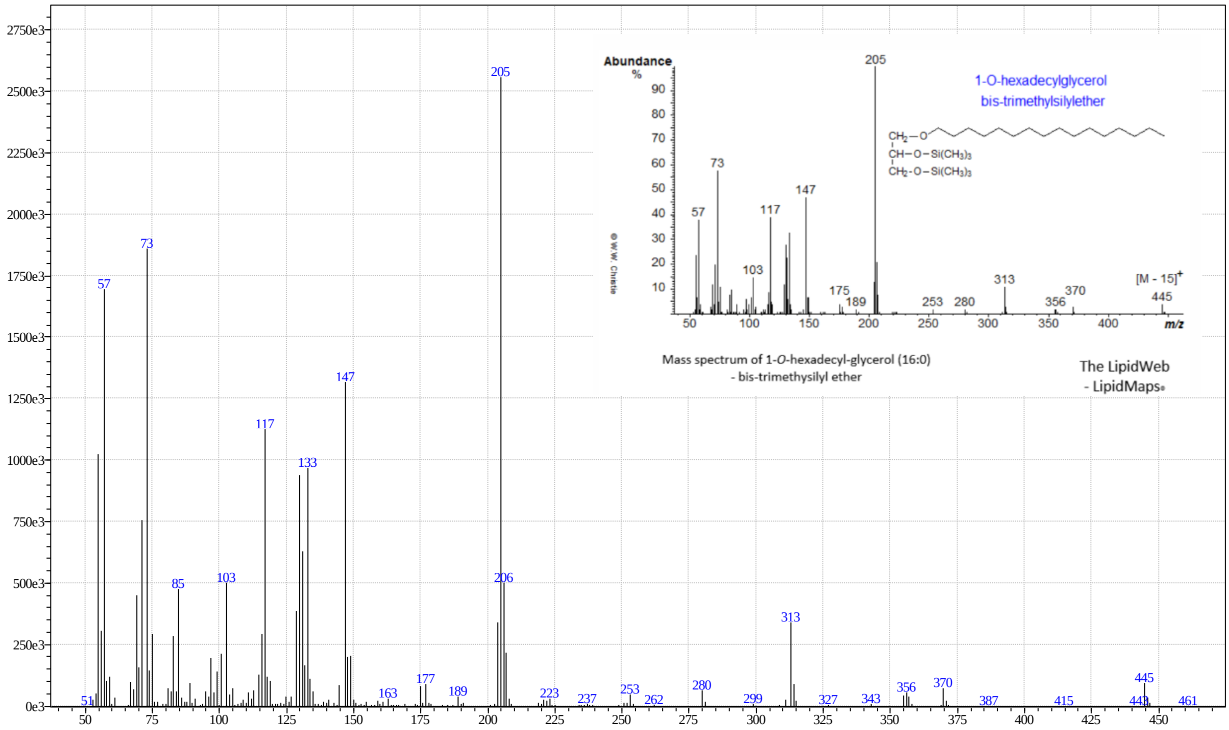

1-*O*-octadecenyl-2,3-*O*-di(trimethylsilyl)-*sn*-glycerol

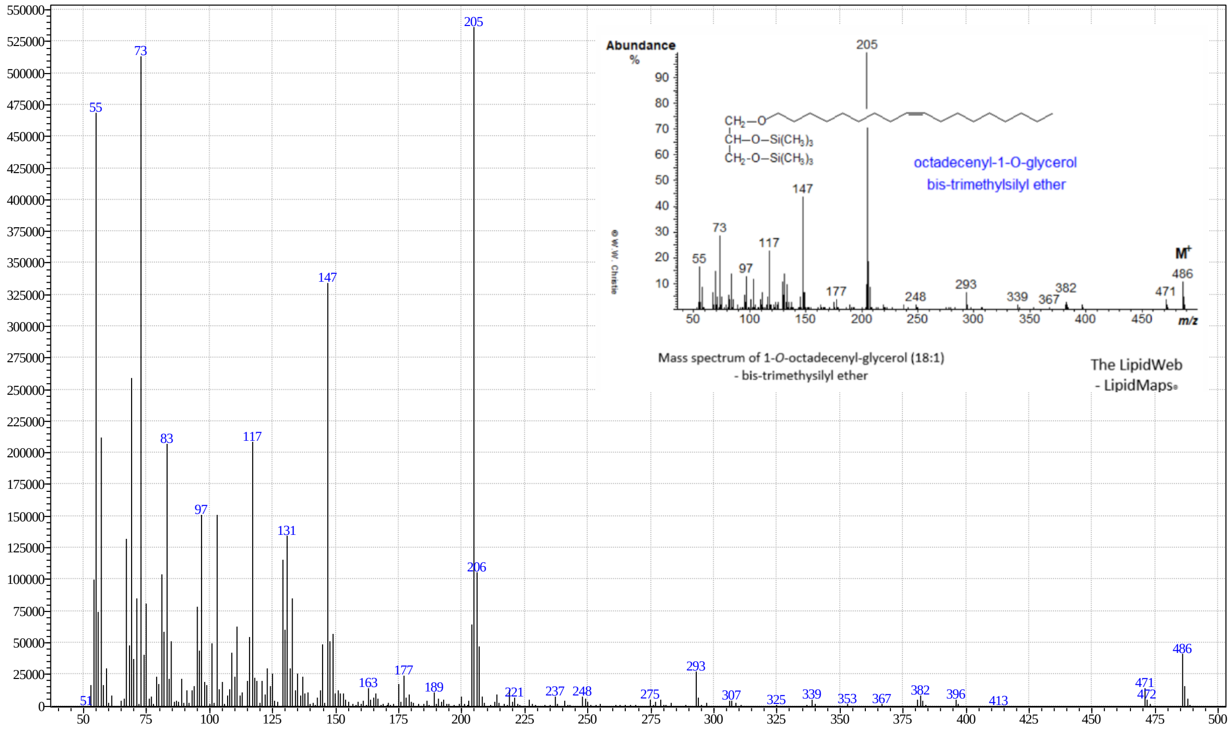

Supplementary Figure S4. Mass spectra of major di-TMS derivatives of 1-*O*-alkyl-*sn*-glycerols obtained by alkaline hydrolysis of DAGE of the soft coral *Paragorgia arborea*. For reference, the correspondence spectrum from the Archive of mass spectra were placed in the upper right corner of each spectrum obtained (Christie, W.W. The LipidWeb. Archive of mass spectra. (2021) https://www.lipidmaps.org/resources/lipidweb/index.php?page=ms/others/others-arch/index1.htm).

Supplementary Figure S5. Examples of DAGE mass spectra. The fraction of diacylglycerol ethers (DAGE) of the soft coral *Paragorgia arborea* was analyzed using reverse-phase liquid chromatography and high resolution tandem mass spectrometry at atmospheric pressure chemical ionization (APCI) in positive (C+) mode. (**A**) Chromatograms of a total ion current (TIC) and four selected molecular ions (*m/z* 811.7160, 865.7610, 919.8070, and 951.8710). The next sections show the observed and calculated masses (*m/z*) of molecular ions, brutto-formulars, double bond equivalent (DBE), differences between observed and calculated masses, the mass spectra at the peak top and MS/MS spectra of the target ions, the predicted structures, and the product ions (*m/z*) of (**B**) DAGE 16:0e/20:5/18:1, (**C**) DAGE 16:0e/20:5/14:0, (**D**) DAGE 16:0e/24:6/18:1, and (**E**) DAGE 16:0e/20:4/24:1. The name of each DAGE molecular species shows the alkyl and acyl groups (number of carbon atoms : number of double bonds) at positions *sn*-1/*sn*-2/*sn*-3 of a glycerol ether backbone.

A.

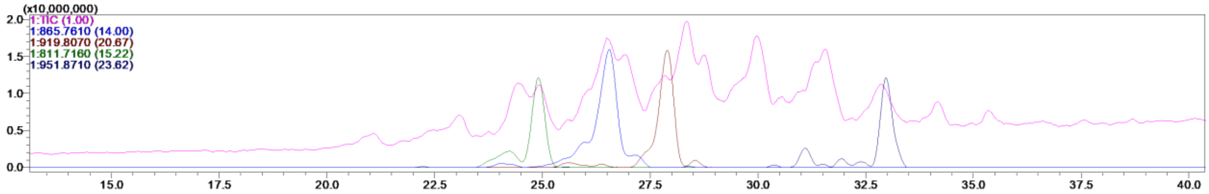

B. DAGE 16:0e/20:5/18:1

M+H	Mass	Formula	DBE	Diff	Diff (ppm)	Diff (mDa)
865.7610	864.7571	C57 H100 O5	8	0.00335	3.88	3.4

Event#: 1 MS(C+)

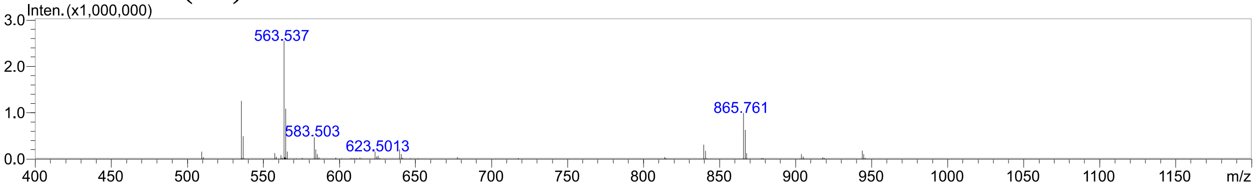

Event#: 2 MS/MS(C+) Precursor : 865.766

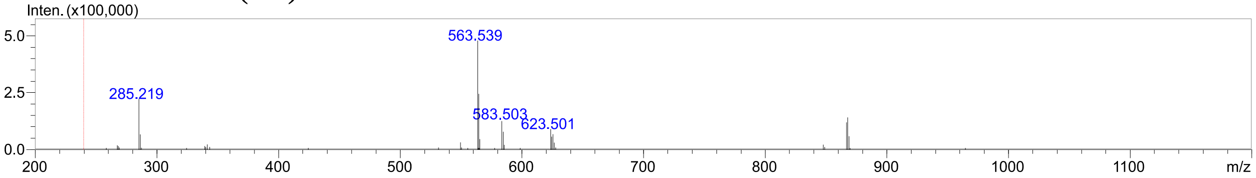

MS	MS2	Loss		
865.761	**563.539**	302.222	20:5//	
	583.503	282.258	18:1//	
	623.5013	242.2597	16:0e	C16 H34 O
	285.2193			C20 H28 O/7

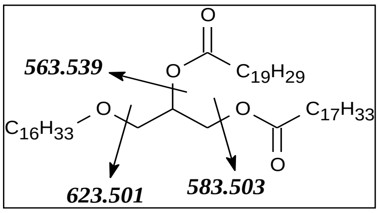

C. DAGE 16:0e/20:5/14:0

M+H	Mass	Formula	DBE	Diff	Diff (ppm)	Diff (mDa)
811.7160	810.7101	C53 H94 O5	7	0.0014	1.73	1.4

Event#: 1 MS(C+)

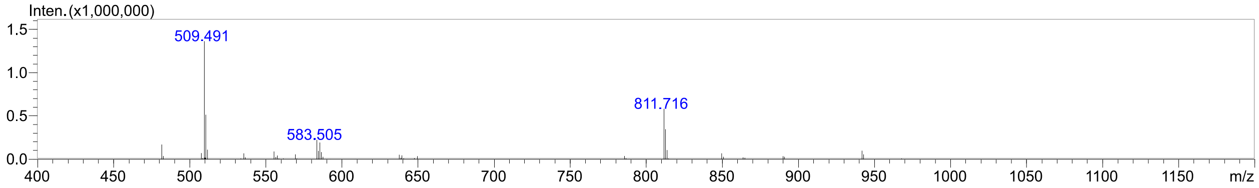

Event#: 2 MS/MS(C+) Precursor : 811.7165

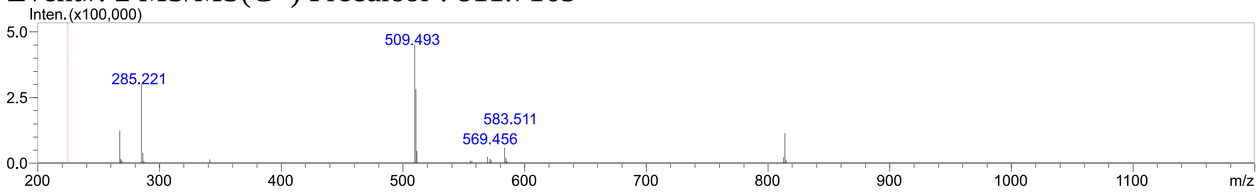

MS	MS2	Loss		
811.7149	**509.493**	302.2219	20:5//	
	583.511	228.2039	14:0//	
	569.456	242.2589	16:0e	C16 H34 O
	285.221			C20 H28 O/7

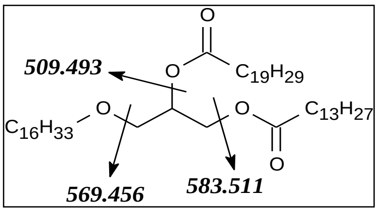

D. DAGE 16:0e/24:6/18:1

M+H	Mass	Formula	DBE	Diff	Diff (ppm)	Diff (mDa)
919.807	918.804	C61 H106 O5	9	0.0043	4.68	4.3

Event#: 1 MS(C+)

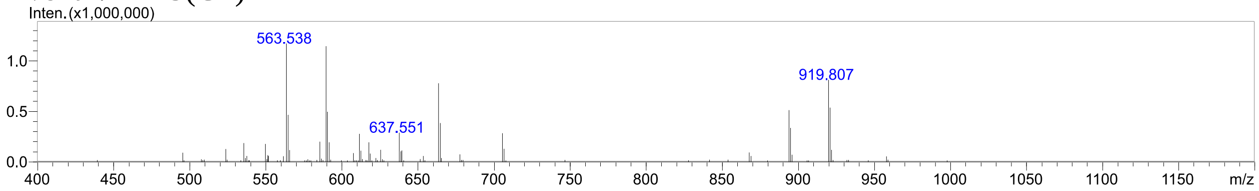

Event#: 2 MS/MS(C+) Precursor : 919.8157

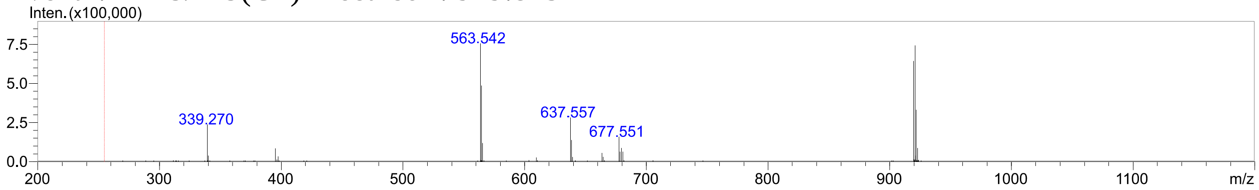

MS	MS2	Loss		
919.807	**563.542**	356.265	24:6//	C24 H36 O2
	637.557	282.25	18:1//	C18 H34 O2
	677.551	242.256	16:0e	C16 H34 O
	339.27			C24 H34 O
	285.221			C20 H28 O/7

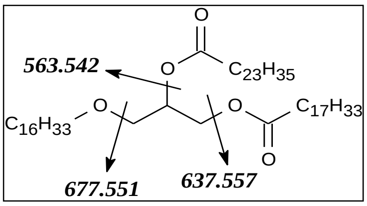

E. DAGE 16:0e/20:4/24:1

M+H	Mass	Formula	DBE	Diff	Diff (ppm)	Diff (mDa)
951.871	950.8666	C63 H114 O5	7	0.0029	3.05	2.9

Event#: 1 MS(C+)

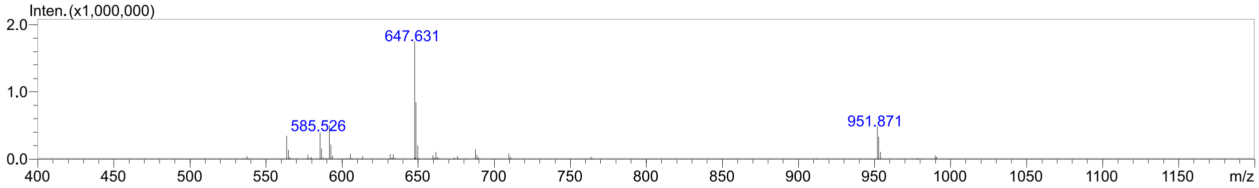

Event#: 2 MS/MS(C+) Precursor : 951.876

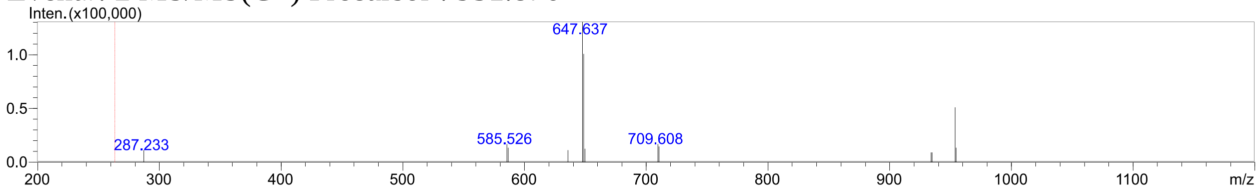

MS	MS2	Loss		
951.871	**647.6315**	304.2395	20:4//	
	585.5221	366.3489	24:1//	C24 H46 O2
	709.608	242.263	16:0e	C16 H34 O
	287.233			C20 H30 O

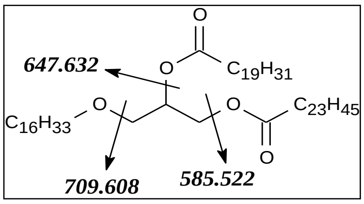

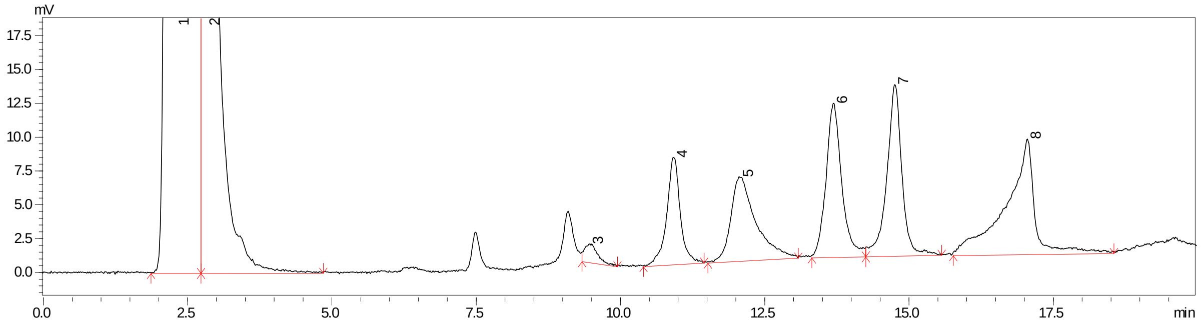

Supplementary Figure S6. Analysis of polar lipid classes. Separation of ceramide aminoethylphosphonate (CAEP) and glycerophospholipids (GPL) of the soft coral *Paragorgia arborea* by high-performance liquid chromatography in HILIC mode with ELSD detection. Peak numbers: 1, triacylglycerol; 2, cholesterol; 3, inositol GPL (PI); 4, ethanolamine GPL (PE); 5, serine GPL (PS); 6, CAEP; 7, choline GPL (PC); 8, system peak.

Supplementary Table S2. Molecular species of diacylglycerol ethers (DAGE). Obtained and calculated molecular ions [M+H]+ (*m/z*), brutto-formulas, characteristic ions (*m/z*), peak areas, retention time on HPLC column (min), and content (% of total, mean ± SD, *n* = 6) of 1-*O*-alkyl-2,3-diacyl-*sn*-glycerols in the soft coral *Paragorgia arborea*. The name of each molecular species shows the alkyl and acyl groups (number of carbon atoms : number of double bonds) at positions *sn*-1/*sn*-2/*sn*-3 of a glycerol ether backbone.

M+H	MW(+)	Brutto-formula	R-Alkyl	R-Acyl (sn-2)	R-Acyl (sn-3)	Molecular species (*sn*-1/*sn*-2/*sn*-3)	RT (min)	S1	S2	S3	S4	S5	S6	Content, %
811.7237	810.7165	C53H94O5	569.4601	509.4955	583.5109	16:0e/20:5/14:0	25.753	8443997	18513485	2711882	10258288	7733655	15299183	2.6 ± 1.1
813.7306	812.7234	C53H96O5	571.4687	509.4875	585.5164	16:0e/20:4/14:0	28.093	8899556	12675471	2750115	18249241	12135521	13390603	2.9 ± 1.2
837.7366	836.7294	C55H96O5	623.5069	535.5102	555.4809	14:0e/20:5/18:1	27.181	9635751	22446968	6380774	12805648	11997116	19666666	3.5 ± 1.0
839.7527	838.7455	C55H98O5	625.5225	535.5107	557.4962	14:0e/20:4/18:1	29.664	8883955	16207212	4602478	18722788	13843502	16408339	3.3 ± 1.0
839.7537	838.7465	C55H98O5	597.4954	537.5261	583.5116	16:0e/20:5/16:0	31.364	10899448	18131623	3908538	18786981	13335830	17439576	3.5 ± 1.2
841.7758	840.7686	C55H100O5	599.5063	537.52	585.5184	16:0e/20:4/16:0	34.257	11242057	16135227	4475248	24829142	17084854	17743316	3.9 ± 1.5
865.769	864.7618	C57H100O5	623.508	563.5421	583.51	16:0e/20:5/18:1	33.132	21444699	33262762	16287436	38196665	33637770	35903224	7.7 ± 1.3
867.7746	866.7674	C57H102O5	625.5147	563.5367	585.5178	16:0e/20:4/18:1	35.849	20054851	29274904	12876087	50342908	37614829	35013060	7.9 ± 2.8
885.7328	884.7256	C59H96O5	643.4718	583.5106	583.5106	16:0e/20:5/20:5	26.859	2744425	11369028	12891897	2338692	11363426	12214500	2.3 ± 1.4
887.7511	886.7439	C59H98O5	645.4901	585.5208	583.5132	16:0e/20:5/20:4	29.305	3877641	17709304	22899148	7165831	21831978	20787312	4.1 ± 2.4
889.7614	888.7542	C59H100O5	647.5004	585.525	585.525	16:0e/20:4/20:4	31.962	3678223	12526733	13347219	8499983	15090501	14553752	2.9 ± 1.1
891.7853	890.7781	C59H102O5	623.5087	589.5588	609.5285	18:1e/20:5/18:1	34.609	6585959	18546889	10886897	8532845	13184044	17644101	3.2 ± 0.8
893.7996	892.7924	C59H104O5	651.53	537.52	637.5506	16:0e/24:6/16:0	38.963	10085392	12863108	6711535	11471073	11525669	13252299	2.9 ± 0.5
893.7996	892.7924	C59H104O5	651.53	591.5669	583.5019	16:0e/20:5/20:1	39.387	6121241	14337988	9376261	18673149	17550342	16776547	3.5 ± 0.8
895.8159	894.8087	C59H106O5	653.5549	591.5746	585.5259	16:0e/20:4/20:1	42.364	15714140	27616804	12887991	33590999	28458939	30035327	6.3 ± 1.3
919.813	918.8058	C61H106O5	677.5475	563.5369	637.551	16:0e/24:6/18:1	40.597	19726841	19834616	14216594	10541893	16887674	20057655	4.6 ± 1.6
921.8301	920.8229	C61H108O5	679.5691	619.6045	583.5123	16:0e/20:5/22:1	45.87	8019933	6050098	4635004	6015354	6916530	6870614	1.8 ± 0.6
921.8302	920.823	C61H108O5	679.5692	563.5416	639.5716	16:0e/24:5/18:1	43.737	7235513	14553805	8950371	9924244	12430569	14734131	2.9 ± 0.3
923.8482	922.841	C61H110O5	681.5854	619.6051	585.526	16:0e/20:4/22:1	49.151	11647637	16870100	13246916	20363103	21502522	20102807	4.5 ± 0.5
939.7801	938.7729	C63H102O5	697.5191	583.5098	637.5588	16:0e/20:5/24:6	33.909	3208138	5019616	12966177	217408	10260943	7628108	1.8 ± 1.5
941.8032	940.796	C63H104O5	701.5579	583.5108	637.5596	16:0e/20:4/24:6	36.664	4160945	7766681	18923607	3031763	16460549	12051705	2.8 ± 2.1
945.8324	944.8252	C63H108O5	677.5428	589.5539	663.5674	18:1e/24:6/18:1	42.133	4428575	6026322	5028977	1419506	4710240	5914754	1.2 ± 0.5
947.8496	946.8424	C63H110O5	705.5886	591.5738	637.5588	16:0e/24:6/20:1	46.924	11162654	9968368	8627482	3963222	8916651	10263471	2.4 ± 1.1
949.8645	948.8573	C63H112O5	735.6348	591.5752	611.5309	14:0e/24:5/22:1	50.427	3550607	9692034	8119851	5233421	9214532	10211291	2.0 ± 0.6
949.8645	948.8573	C63H112O5	681.5879	645.619	611.5309	18:1e/20:4/22:1	50.702	2847787	2327782	2435536	1695975	2832940	2716706	0.7 ± 0.2
951.8674	950.8602	C63H114O5	709.6064	647.6364	585.5265	16:0e/20:4/24:1	58.015	3472514	10673544	6713905	5250310	8124057	10382803	1.9 ± 0.5
975.8692	974.862	C65H114O5	733.6084	619.5986	637.5504	16:0e/24:6/22:1	54.55	7084296	5260176	5919039	1275793	5327809	5681289	1.4 ± 0.8
977.8943	976.8871	C65H116O5	735.6194	619.5971	639.5658	16:0e/24:5/22:1	59.345	3110222	6610436	5674297	1352920	5178572	6493077	1.3 ± 0.5

Supplementary Table S3. Molecular species of polar lipids. Molecular ions [M+H]+ (m/z), brutto-formulas, differences between obtained and calculated masses (ppm), peak areas, retention time on HPLC column (min), and content (% of lipid class and total polar lipids, mean ± SD, *n* = 3) of five major polar lipid classes in the soft coral *Paragorgia arborea*. 1,2-Diacyl groups are abbreviated as X/Y, where X and Y are acyl groups (number of carbon atoms : number of double bonds). Ether phospholipids are abbreviated as Xa/Y or Xp/Y, where Xa is alkyl group and Xp is alk-1-enyl group at position *sn*-1 of a glycerol ether backbone, and Y is acyl group at position *sn*-2. CAEP molecules are abbreviated as Xd/Y; Xd, sphingoid base; Y, acyl group. The green background indicates the molecular species with the concentration more than 5% of the correspondent lipid class. Trace peaks of molecular species that showed only the fragment ion of the characteristic polar groups are marked by “x”.

Molecular ion, [M+H]+ (*m/z*)	Brutto-formula	Diff (ppm)	Name	Retention time (min)	Sample 1 (area)	Sample 2 (area)	Sample 3 (area)	Content (% of lipid class, mean ± SD)	Average content (% of polar lipids)
Ceramide aminoethylphosphonate (CAEP)									
641.55	C36 H69 N2 O5 P	0.13	20:3d/14:0	13.585	1571977	443894	615756	0.05 ± 0.02	0.01
641.55	C36 H69 N2 O5 P	0.13	19:3d/15:0	14.722	2773373	124533	1937426	0.06 ± 0.04	0.02
641.55	C36 H69 N2 O5 P	0.13	18:3d/16:0	15.459	26015349	3986322	15503796	0.73 ± 0.06	0.21
643.55	C36 H71 N2 O5 P	1.19	18:2d/16:0	16.438	68510067	15867123	87655557	2.94 ± 0.98	0.87
643.55	C36 H71 N2 O5 P	0.1	18:2d/16:0	17.184	254282422	37062049	135911939	6.78 ± 0.83	1.99
645.55	C36 H73 N2 O5 P		18:1d/16:0	18.452	11137488	374839	7130723	0.24 ± 0.15	0.07
645.55	C36 H73 N2 O5 P		18:1d/16:0	19.294	9801580	1207878	5034225	0.25 ± 0.05	0.07
647.55	C36 H75 N2 O5 P		18:0d/16:0	21.182	2315324	241077	824176	0.05 ± 0.02	0.01
655.55	C37 H71 N2 O5 P	4.03	19:3d/16:0	16.015	28726191	4944984	19298809	0.87 ± 0.02	0.26
655.55	C37 H71 N2 O5 P	1.74	19:3d/16:0	17.22	613221175	131726104	448104900	20.62 ± 2.01	6.06
657.55	C37 H73 N2 O5 P	1.54	19:2d/16:0	19.066	732195364	111878004	417520462	20.22 ± 1.79	5.94
659.6	C37 H75 N2 O5 P	2.64	19:1d/16:0	20.911	65631624	10214982	35010442	1.79 ± 0.20	0.53
661.6			x	22.558	1768644	143266	740076	0.04 ± 0.01	0.01
667.6	C38 H71 N2 O5 P		20:4d/16:0	15.538	1350211	246163	576151	0.04 ± 0.01	0.01
669.55	C38 H73 N2 O5 P	1.62	20:3d/16:0	18.512	392013585	57737160	228119250	10.77 ± 1.03	3.17
669.55	C38 H73 N2 O5 P		20:3d/16:0	19.94	782815	1766949	6126164	0.20 ± 0.16	0.06
671.6	C38 H75 N2 O5 P	1.59	20:2d/16:0	20.449	93285090	22323219	121173197	4.08 ± 1.37	1.20
671.6	C38 H75 N2 O5 P	1.44	20:2d/16:0	21.121	265914844	41226952	143732470	7.26 ± 0.77	2.13
671.6	C38 H75 N2 O5 P		20:2d/16:0	22.046	15291422	895049	5239048	0.29 ± 0.16	0.08
673.6	C38 H77 N2 O5 P	0.76	20:1d/16:0	22.594	44620798	8058869	25339923	1.30 ± 0.12	0.38
673.6	C38 H77 N2 O5 P		20:1d/16:0	23.454	4641249	310101	1747151	0.09 ± 0.05	0.03
683.6	C39 H75 N2 O5 P		21:3d/16:0	20.164	3168799	1797635	4889885	0.21 ± 0.11	0.06
683.6	C39 H75 N2 O5 P	3.86	21:3d/16:0	20.975	9966037	1765762	6104046	0.30 ± 0.01	0.09
683.6	C39 H75 N2 O5 P		21:3d/16:0	21.71	4058856	277419	1792430	0.08 ± 0.04	0.02

685.6	C39 H77 N2 O5 P		21:2d/16:0	21.962	1735831	1289300	883289	0.11 ± 0.10	0.03
685.6	C39 H77 N2 O5 P		21:2d/16:0	22.37	10294025	703598	6588063	0.25 ± 0.11	0.07
685.6	C39 H77 N2 O5 P	5.28	21:0d/16:1	23.16	18069062	1519505	10607247	0.43 ± 0.15	0.13
687.6	C39 H79 N2 O5 P		21:0d/16:0	24.283	10508292	1920472	7159739	0.33 ± 0.01	0.10
695.6	C40 H75 N2 O5 P		22:4d/16:0	19.523	766067	102919	406510	0.02 ± 0.00	0.01
695.6	C40 H75 N2 O5 P	3.65	22:4d/16:0	20.332	14684386	2772572	18175277	0.59 ± 0.21	0.17
697.6	C40 H75 N2 O5 P	1.7	22:3d/16:0	21.86	28076337	4249480	15337304	0.76 ± 0.08	0.22
697.6	C40 H77 N2 O5 P	2.6	22:3d/16:0	22.784	45607629	7274893	27266097	1.30 ± 0.08	0.38
699.55	C40 H79 N2 O5 P	3.38	22:2d/16:0	23.967	473246711	93648865	332105927	15.24 ± 0.86	4.48
699.55	C40 H79 N2 O5 P		22:2d/16:0	24.527	844731	113408	1314795	0.04 ± 0.02	0.01
699.55	C40 H79 N2 O5 P		22:2d/16:0	24.905	263145	27178	315089	0.01 ± 0.00	0.00
701.6	C40 H81 N2 O5 P		22:1d/16:0	24.984	4331948	8636574	27038197	0.95 ± 0.72	0.28
709.6			x	22.599	496783	73669	110827	0.01 ± 0.01	0.00
709.6			x	23.516	2446867	209129	1555841	0.06 ± 0.02	0.02
711.6			x	24.97	4278590	433630	2880674	0.11 ± 0.03	0.03
713.65	C41 H83 N2 O5 P		22:2d/17:0	25.194	6564154	919680	4160743	0.18 ± 0.02	0.05
721.6	C42 H77 N2 O5 P		22:4d/18:1	21.196	680045	30812	537463	0.02 ± 0.01	0.00
727.6	C42 H83 N2 O5 P	2.12	24:2d/16:0	26.132	10228912	2242960	7374629	0.35 ± 0.04	0.10
Ethanolamine glycerophospholipids (PE)									
704.5	C39 H79 N O7 P		16:0a/18:1	25.989	1196886	401901	1162055	0.40 ± 0.04	0.06
710.5			x	20.304	135664	60919	106430	0.05 ± 0.02	0.01
710.5			x	20.819	324501	63615	241009	0.08 ± 0.02	0.01
712.5	C39 H71 N O8 P		14:0/20:4	22.492	581415	179949	613711	0.19 ± 0.00	0.03
712.5	C39 H71 N O8 P		14:1/20:3	23.12	664941	150862	604089	0.19 ± 0.03	0.03
722.5			x	19.643	401301	55168	139743	0.08 ± 0.05	0.01
722.5			x	20.131	158610	60525	127376	0.05 ± 0.01	0.01
722.5			x	20.886	620541	154768	314989	0.16 ± 0.06	0.02
722.5			x	21.253	260636	72084	68619	0.06 ± 0.04	0.01
722.5	C41 H72 N O7 P	5.8	16:0p/20:5	22.446	5120784	1946518	6446455	1.95 ± 0.20	0.30
724.5			x	21.435	178181	386710.5	773421	0.24 ± 0.18	0.04
724.5	C41 H74 N O7 P		16:1a/20:5	22.087	2199361	781455	1309300	0.67 ± 0.24	0.10
724.5	C41 H74 N O7 P	1.06	16:1a/20:4	23.004	12169314	3610641	15067635	4.24 ± 0.37	0.65
724.5	C41 H74 N O7 P	7.09	16:0p/20:4	24.27	31793057	9455883	33525513	10.49 ± 0.25	1.62
726.6	C41 H76 N O7 P	3.15	16:0a/20:4	24.692	38179477	12774984	51309272	14.26 ± 1.47	2.20
728.6	C41 H78 N O7 P	22.04	36:3a	25.555	821788	94014	845337	0.21 ± 0.10	0.03
730.6			x	26.421	504638	303906	583963	0.23 ± 0.09	0.04
732.6			x	28.032	349652	45079	173170	0.07 ± 0.04	0.01
736.6			x	24.302	492354	120780	427555	0.14 ± 0.02	0.02

738.6			x	21.52	745120	289000	577633	0.25 ± 0.07	0.04
738.6			x	23.841	638590	68100	437101	0.14 ± 0.07	0.02
738.6	C42 H76 N O7 P	6.1	37:5a	24.267	845694	239482	685261	0.25 ± 0.04	0.04
738.6	C42 H76 N O7 P	3.5	17:0p/20:4	24.921	2057199	647675	2521726	0.73 ± 0.04	0.11
738.6	C42 H76 N O7 P	7.3	17:0p/20:4	25.542	3592348	922570	3691217	1.12 ± 0.11	0.17
740.6			x	25.501	1231106	172675	1157624	0.32 ± 0.12	0.05
740.6			x	25.769	449050	46325	80892	0.08 ± 0.07	0.01
748.6	C43 H74 N O7 P	18.51	18:2p/20:4	21.954	147593	32334	54321	0.03 ± 0.02	0.01
748.6	C43 H74 N O7 P		18:1p/20:5	22.872	144105	23609	52929	0.03 ± 0.02	0.00
750.6	C43 H76 N O7 P	2.78	18:1a/20:5	23.574	3787479	865149	4385168	1.20 ± 0.22	0.18
750.6			x	24.495	357128	99987	635531	0.14 ± 0.05	0.02
750.6	C43 H76 N O7 P		18:1p/20:4	24.779	3064488	1197953	4926632	1.29 ± 0.24	0.20
750.6	C43 H76 N O7 P		18:0p/20:5	25.19	174860	52504	53948	0.04 ± 0.02	0.01
750.6			x	25.318	141506	129199	481937	0.11 ± 0.06	0.02
752.6			x	24.627	502501	115252	296518	0.13 ± 0.04	0.02
752.6	C43 H78 N O7 P	4.43	18:1a/20:4	25.107	17168878	4930133	18370757	5.63 ± 0.22	0.87
752.6	C43 H78 N O7 P	11.75	18:0a/20:5	25.644	987339	804434	2583674	0.67 ± 0.29	0.10
752.6			x	25.781	2597445	196250	387121	0.40 ± 0.41	0.06
752.6	C43 H78 N O7 P	2.97	18:0p/20:4	26.526	1447945	298400	920629	0.37 ± 0.11	0.06
754.6			18:0a/20:4	26.784	1723257	579611	2328990	0.65 ± 0.07	0.10
764.6			x	24.878	311949	135259	565195	0.14 ± 0.03	0.02
764.6			x	25.219	765308	169301	661973	0.22 ± 0.04	0.03
764.6			x	25.804	584468	95452	720401	0.18 ± 0.06	0.03
766.6	C44 H80 N O7 P	0.89	39:5a	26.097	999573	194925	1065081	0.29 ± 0.07	0.05
766.6			x	26.513	529553	225181	788647	0.22 ± 0.04	0.03
766.6			x	27.133	349493	249885	431375	0.18 ± 0.09	0.03
768.6			x	23.401	1069153	206599	788018	0.28 ± 0.07	0.04
776.6	C45 H78 N O7 P		16:1a/24:6	24.27	1464268	415278	1238369	0.44 ± 0.06	0.07
776.6	C45 H78 N O7 P	2.41	16:0p/24:6	25.691	6074665	2253818	7796948	2.31 ± 0.22	0.36
778.6	C45 H80 N O7 P		16:0a/24:6	25.563	1934078	430864	1983699	0.58 ± 0.10	0.09
778.6	C45 H80 N O7 P	2.04	16:1a/24:5	25.987	28037712	8436342	30623223	9.40 ± 0.15	1.45
778.6	C45 H80 N O7 P	0.15	16:0p/24:5	26.751	12090679	3669615	14457485	4.19 ± 0.24	0.65
780.6	C45 H82 N O7 P	0.43	16:0a/24:5	27.105	31546409	11089511	33635773	11.07 ± 0.93	1.71
780.6	C45 H82 N O7 P	2.48	16:0p/24:4	27.647	987689	98916	130113	0.16 ± 0.15	0.02
782.6			x	23.547	799013	75716	149311	0.13 ± 0.12	0.02
782.6			x	24.193	588193	70910	131317	0.11 ± 0.08	0.02
782.6			x	24.925	1105393	114704	705130	0.24 ± 0.13	0.04
790.6			x	23.711	179956		68225	0.03 ± 0.03	0.00

790.6	C46 H80 N O7 P	12.69	41:7a	25.648	236821	30000	135781	0.05 ± 0.03	0.01
790.6	C46 H80 N O7 P	7.62	41:7a	25.922	389870	46452	177593	0.08 ± 0.05	0.01
790.6	C46 H80 N O7 P	3.02	41:7a	26.124	708898	13808	125296	0.10 ± 0.12	0.02
790.6	C46 H80 N O7 P		17:0p/24:6	26.369	234730	189245	1074319	0.21 ± 0.13	0.03
790.6	C46 H80 N O7 P	1.03	17:0p/24:6	26.736	1471904	428894	1542243	0.48 ± 0.02	0.07
792.6			x	25.191	202286	133039	167566	0.09 ± 0.05	0.01
792.6			x	25.426	400689	88946	178279	0.10 ± 0.04	0.01
792.6	C46 H82 N O7 P	3.71	41:6a	26.479	717343	172531	404836	0.19 ± 0.06	0.03
792.6	C46 H82 N O7 P	8.51	17:1a/24:5	26.751	887723	319164	734536	0.29 ± 0.06	0.05
792.6	C46 H82 N O7 P	0.46	17:0p/24:5	27.281	3066656	1769817	3397626	1.34 ± 0.52	0.21
792.6	C46 H82 N O7 P	0.46	17:0p/24:5	27.882	5329340	1784837	6321431	1.90 ± 0.08	0.29
794.6			x	26.49	164445	119421	394538	0.10 ± 0.04	0.02
794.6			x	27.781	887897	470234	1635210	0.44 ± 0.12	0.07
794.6			x	27.982	474833	175036	41050	0.12 ± 0.10	0.02
794.6			x	28.092	698967	13406	41050	0.09 ± 0.13	0.01
802.6				25.016	455220	55109	247816	0.10 ± 0.05	0.01
802.6	C47 H80 N O7 P		18:1p/24:6	26.091	1042101	253668	1360347	0.35 ± 0.07	0.05
804.6			x	25.118	737469	109015	477399	0.17 ± 0.07	0.03
804.6	C47 H82 N O7 P	1.83	18:1a/24:6	26.304	15637636	5244968	18845881	5.62 ± 0.28	0.87
804.6	C47 H82 N O7 P		18:1p/24:5	27.19	2048222	548549	2132768	0.65 ± 0.05	0.10
806.6	C47 H84 N O7 P	0.39	18:0a/24:6	27.432	15938488	6041629	18162474	5.88 ± 0.65	0.90
806.6	C47 H84 N O7 P	9.16	18:1a/24:5	28.014	3091495	433193	2882429	0.80 ± 0.30	0.12
806.6			x	28.546	311126	416413	35159	0.19 ± 0.23	0.03
806.6	C47 H84 N O7 P		18:0p/24:5	28.759	534366	129889	496976	0.16 ± 0.02	0.02
808.6	C47 H86 N O7 P	4.54	18:0a/24:5	29.133	3758161	811192	2893456	1.02 ± 0.22	0.16
810.6			x	25.725	825348	123307	755823	0.22 ± 0.07	0.03
818.6			x	27.629	201141	58941	97968	0.05 ± 0.02	0.01
818.6			x	28.112	381352	62066	420589	0.11 ± 0.04	0.02
818.6			x	28.331	183494	93229	53532	0.06 ± 0.04	0.01
820.6			x	26.682	462212	144484	348331	0.14 ± 0.03	0.02
820.6			x	27.171	247054	63670	110850	0.06 ± 0.03	0.01
822.6	C47 H85 N O8 P		18:0/24:5	27.766	1527016	232611	910158	0.35 ± 0.14	0.05
832.6			x	28.231	462898	329163	579985	0.23 ± 0.11	0.04
834.6	C49 H88 N O7 P	0.22	20:1a/24:5	29.392	1223421	313696	1723035	0.43 ± 0.10	0.07
836.6			x	26.71	183883	173427	376306	0.12 ± 0.06	0.02
836.6			x	31.126	38998	24955.02383	54148	0.01 ± 0.01	0.00
Choline glycerophospholipids (PC)									
706.6			x	21.979	1205627	457820	1540933	0.02 ± 0.00	0.01

712.6	C40 H74 N O7 P		16:1a/16:3	17.404	380148	400863	1083926	0.01 ± 0.01	0.00
716.6	C40 H78 N O7 P		16:0a/16:2	23.178	3373571	673857	1820497	0.04 ± 0.01	0.01
718.6	C40 H80 N O7 P	0.16	14:0a/18:1	24.98	15075635	5127217	14194917	0.22 ± 0.01	0.06
720.6	C40 H82 N O7 P	2.46	16:0a/16:0	27.047	6510070	2680579	8045760	0.11 ± 0.02	0.03
724.6			x	17.348	1073579	222235	989350	0.01 ± 0.00	0.00
726.6	C41 H76 N O7 P	5.63	33:4a	20.173	2762235	546338	2258542	0.03 ± 0.01	0.01
730.6			x	24.374	702657	157624	168466	0.01 ± 0.00	0.00
730.6			x	24.867	588984	202638	754048	0.01 ± 0.00	0.00
730.6			x	25.545	557911	68325	327657	0.01 ± 0.00	0.00
732.6	C40 H78 N O8 P	4.67	16:0/16:1	22.732	10408631	3097793	10522179	0.15 ± 0.01	0.04
734.6			x	25.315	3328844	741186	2905744	0.04 ± 0.01	0.01
734.6			x	26.837	408199	114740	458617	0.01 ± 0.00	0.00
736.6	C42 H74 N O7 P	1.31	x	17.172	15274743	4995087	14914353	0.22 ± 0.00	0.06
736.6			x	18.595	129890	149626	747876	0.01 ± 0.00	0.00
738.6			x	19.111	2674534	564114	2887142	0.04 ± 0.01	0.01
738.6	C42 H76 N O7 P	1.06	14:1a/20:4	20.094	206103863	69191109	209451797	3.07 ± 0.10	0.86
740.6	C42 H78 N O7 P	1.04	14:0a/20:4	22.526	624434915	209269202	523538362	8.73 ± 0.73	2.45
742.6	C42 H80 N O7 P	0.02	14:0a/20:3	24.038	12273494	2576095	4815289	0.12 ± 0.05	0.03
744.6	C42 H82 N O7 P	3.32	16:1a/18:1	25.844	15735892	3394410	8528130	0.17 ± 0.05	0.05
746.6	C42 H84 N O7 P	1.1	16:0a/18:1	27.389	43766306	15228375	40626091	0.64 ± 0.03	0.18
750.6	C43 H76 N O7 P	1.49	35:6a	19.092	2674366	1716831	5458183	0.07 ± 0.02	0.02
750.6			x	19.37	0	2322059	5423885	0.06 ± 0.05	0.02
750.6			x	20.164	0	493856	1350996	0.01 ± 0.01	0.00
752.6	C43 H78 N O7 P	0.04	35:5a	21.807	22887857	9026782	3012511	0.26 ± 0.19	0.07
752.6	C43 H78 N O7 P	1.69	15:0a/20:5	22.409	38282192	14043722	11609649	0.45 ± 0.24	0.13
754.6	C43 H80 N O7 P	1.3	35:4a	23.885	35211018	15207867	42553441	0.61 ± 0.09	0.17
754.6	C43 H80 N O7 P	1.84	15:0a/20:4	24.362	98283037	28887779	76343420	1.28 ± 0.13	0.36
758.6			x	27.327	1169507	50837	1159006	0.01 ± 0.01	0.00
758.6	C43 H84 N O7 P		35:2a	28.181	5603365	1681247	5360172	0.08 ± 0.00	0.02
760.6	C42 H82 N O8 P	1.6	16:0/18:1	25.703	43409874	14638231	37376518	0.61 ± 0.04	0.17
764.6	C44 H78 N O7 P	0.04	16:1a/20:5	21.09	38752615	12514964	36939958	0.56 ± 0.00	0.16
764.6	C44 H78 N O7 P	1.92	36:6a	22.256	32573775	13773640	32743669	0.52 ± 0.08	0.15
766.6	C44 H80 N O7 P	0.24	16:1a/20:4	23.39	89830776	34674334	100791196	1.45 ± 0.14	0.41
766.6	C44 H80 N O7 P	1.59	16:0a/20:5	24.27	783679850	330657467	952095680	13.45 ± 1.87	3.78
766.6	C44 H80 N O7 P	2.2	16:0a/20:5	25.235	78502281	15889500	37307564	0.80 ± 0.30	0.22
768.6	C44 H82 N O7 P		16:0a/20:4			18403922	51181778	0.53 ± 0.46	0.15
768.6	C44 H82 N O7 P	1.65	16:0a/20:4	25.755	2181659130	608964524	2035239346	29.73 ± 2.36	8.35
780.6	C44 H78 N O8 P	1.39	16:0/20:5	21.73	207041399	66118879	207333956	3.02 ± 0.10	0.85

782.6	C44 H80 N O8 P	0.17	16:0/20:4	23.851	233511879	68229990	226357719	3.27 ± 0.21	0.92
786.6	C44 H84 N O8 P	0.8	18:1/18:1	26.122	12364560	5046301	8330190	0.18 ± 0.05	0.05
788.6	C44 H86 N O8 P	5.86	18:0/18:1	25.68	21157204	2638555	7019004	0.18 ± 0.11	0.05
788.6	C44 H86 N O8 P	2.14	36:1	28.019	10813514	3178732	11217346	0.16 ± 0.01	0.04
792.6	C46 H82 N O7 P	1.73	18:1a/20:5	24.703	203332565	70142231	189446430	2.97 ± 0.13	0.83
792.6	C46 H82 N O7 P	2.23	16:0a/22:6	25.508	78379773	21309036	59923316	0.99 ± 0.12	0.28
794.6	C46 H84 N O7 P	2.54	18:1a/20:4	26.026	388694277	133789735	358499952	5.65 ± 0.27	1.59
794.6	C46 H84 N O7 P	0.15	18:0a/20:5	26.778	83173233	25055521	66912272	1.11 ± 0.09	0.31
796.6	C46 H86 N O7 P	1.97	38:4a	27.54	14478521	7351858	20618151	0.28 ± 0.06	0.08
796.6	C46 H86 N O7 P	2.1	18:0a/20:4	28.096	95023040	30596038	85207472	1.34 ± 0.04	0.38
806.6	C46 H80 N O8 P	5.13	18:1/20:5	22.432	35412801	13436621	39699884	0.57 ± 0.05	0.16
806.6	C46 H80 N O8 P	3.27	16:0/22:6	23.592	14579991	4998836	14026891	0.21 ± 0.01	0.06
808.8	C46 H82 N O8 P	4.68	18:1/20:4	24.432	40088362	13676972	37745207	0.58 ± 0.02	0.16
808.8	C46 H82 N O8 P	0.84	18:0/20:5	25.227	35668936	13269198	35003774	0.54 ± 0.04	0.15
810.8	C46 H84 N O8 P	9.67	18:1/20:3	25.917	12792808	6852785	17523176	0.25 ± 0.06	0.07
810.8	C46 H84 N O8 P	3.25	18:0/20:4	26.439	48935785	15485867	41913149	0.68 ± 0.04	0.19
814.8	C46 H88 N O8 P	1.93	18:1/20:1	28.359	10844853	3721842	10177968	0.16 ± 0.01	0.04
816.8	C46 H90 N O8 P	1.45	18:0/20:1	30.305	2955229	564231	2861409	0.04 ± 0.01	0.01
818.8	C48 H84 N O7 P	2.83	39:0a	25.282	12071915	4040996	11652664	0.18 ± 0.00	0.05
818.8	C48 H84 N O7 P	7.24	39:0a	25.848	12731980	7004397	12840644	0.23 ± 0.07	0.06
820.8	C48 H86 N O7 P	6.43	40:6a	26.595	6759869	3346785	10649350	0.14 ± 0.03	0.04
820.8	C48 H86 N O7 P	1.18	16:0a/24:6	27.076	168376990	62442265	171381433	2.59 ± 0.17	0.73
822.8	C48 H88 N O7 P		18:0a/22:5	27.066	46698390	1068577	3185272	0.26 ± 0.36	0.07
822.8	C48 H88 N O7 P	2.33	16:0a/24:5	28.359	114364746	38679332	115297383	1.70 ± 0.05	0.48
824.8	C47 H86 N O8 P	14.18	39:4	24.648	3032714	205801	1471766	0.03 ± 0.02	0.01
824.8	C47 H86 N O8 P	5.56	39:4	25.188	19898494	1251030	16862976	0.20 ± 0.13	0.06
824.8	C48 H90 N O7 P	11.86	40:4a	30.499	1581467	73247	1824943	0.02 ± 0.01	0.01
826.8			x	25.827	2807799	633962	1420269	0.03 ± 0.01	0.01
832.8	C48 H82 N O8 P	1.66	40:7	23.72	3218640	1280594	4243020	0.06 ± 0.01	0.02
832.8	C48 H82 N O8 P	13.57	40:7	27.098	4805145	665124	2545887	0.05 ± 0.02	0.01
832.8	C49 H86 N O7 P	1.16	41:7a	27.84	20877558	7422567	22278371	0.32 ± 0.02	0.09
834.8	C48 H84 N O8 P	7.12	16:0/24:6+20:1/20:5	25.511	171824852	52237928	150049658	2.35 ± 0.11	0.66
834.8	C49 H88 N O7 P	2.54	41:6a	29.119	25154198	3732158	27830205	0.32 ± 0.13	0.09
838.8	C48 H88 N O8 P		40:4	26.752	27250155	11218965	31178527	0.45 ± 0.05	0.13
838.8	C48 H88 N O8 P	2.47	40:4	28.27	4288402	587039	3277074	0.05 ± 0.02	0.01
838.8	C48 H88 N O8 P	3.5	40:4	28.906	5005184	1826903	2530120	0.06 ± 0.02	0.02
840.8	C50 H82 N O7 P	2.94	41:3a	23.223	2441370	843573	1726119	0.03 ± 0.01	0.01

842.8	C48 H92 N O8 P	4.43	18:1/22:1	30.528	2011067	427667	1025378	0.02 ± 0.01	0.01
846.8	C50 H88 N O7 P	1.32	18:1a/24:6	27.408	26259610	7688227	24155633	0.36 ± 0.02	0.10
848.8	C49 H86 N O8 P	4.7	41:6	24.106	23966871	7742574	23483677	0.35 ± 0.01	0.10
848.8	C49 H86 N O8 P	29.12	41:6	26.27	4308368	302230	216929	0.03 ± 0.03	0.01
848.8	C49 H86 N O8 P	19.1	41:6	26.68	4303742	177135	817460	0.03 ± 0.03	0.01
848.8	C50 H90 N O7 P	2.32	18:1a/24:5	28.713	11085155	4729938	11628275	0.18 ± 0.03	0.05
848.8	C50 H90 N O7 P	2.96	18:1a/24:5	29.335	6509588	1916080	6467678	0.09 ± 0.01	0.03
850.6	C49 H88 N O8 P	6.98	41:5	25.556	44115541	14482971	33392535	0.59 ± 0.08	0.17
850.6	C50 H92 N O7 P	1.67	18:0a/24:5	30.49	9418191	3189309	9371124	0.14 ± 0.00	0.04
852.6			x	18.689	3063982	1557156	4313480	0.06 ± 0.01	0.02
852.6	C49 H90 N O8 P	9.02	41:4	26.79	7922194	2969405	8393353	0.12 ± 0.01	0.03
858.8	-		x	31.252	900759	833225	2125919	0.03 ± 0.01	0.01
860.8	C50 H86 N O8 P	8.01	18:1/24:6	25.902	20624501	7939587	23575958	0.34 ± 0.03	0.09
860.8	C50 H86 N O8 P	21	18:0/24:5	26.738	1671213	893553	636847	0.02 ± 0.02	0.01
862.8	C50 H88 N O8 P	2.24	18:1/24:5	27.189	11359107	4370773	13133001	0.19 ± 0.02	0.05
862.8	C50 H88 N O8 P	1.66	18:0/24:6	27.785	25773312	7777722	26834325	0.37 ± 0.03	0.11
864.8	C50 H90 N O8 P	9.24	42:5	28.513	1877340	879655	3016068	0.04 ± 0.01	0.01
864.8	C50 H90 N O8 P	1.6	18:0/24:5	29.101	19350340	7647303	20685905	0.31 ± 0.03	0.09
868.8	C51 H82 N O8 P	3.9	42:7	18.556	2800826	1096531	2560779	0.04 ± 0.01	0.01
870.8	C51 H84 N O8 P	1.99	42:8	21.432	10138762	3268405	9825469	0.15 ± 0.00	0.04
872.8	C51 H86 N O8 P	1.93	42:1	23.59	1914998	876069	2417728	0.03 ± 0.01	0.01
872.8			x	23.961	864337	91605	212337	0.01 ± 0.01	0.00
874.8	C51 H88 N O8 P	16.86	42:0	27.128	847330	350704	316644	0.01 ± 0.01	0.00
874.8			x	27.847	564299	129410	305208	0.01 ± 0.00	0.00
874.8	C52 H92 N O7 P	0.71	44:7a	29.637	3172281	1067654	2395487	0.04 ± 0.01	0.01
876.8	C51 H90 N O8 P	1.92	19:0/24:6	26.483	5686214	2018719	5605267	0.09 ± 0.00	0.02
876.8	C52 H94 N O7 P	1.33	44:6a	31.002	1841823	717213	2206149	0.03 ± 0.00	0.01
878.8	C51 H92 N O8 P	7.44	43:7	27.795	4358394	1439646	5142784	0.07 ± 0.01	0.02
884.8			x	25.451	958986	96951	166681	0.01 ± 0.01	0.00
886.8	C52 H88 N O8 P		20:3/24:5	26.66	1909713	708107	2169897	0.03 ± 0.00	0.01
886.8			x	27.534	1850263	678530	2388036	0.03 ± 0.00	0.01
888.8	C52 H90 N O8 P	1.33	20:1/24:6	28.029	20046131	6531569	20834581	0.30 ± 0.01	0.08
888.8			x	28.725	217743	184865	641947	0.01 ± 0.00	0.00
890.8	C52 H92 N O8 P	1.5	20:1/24:5	29.293	11535141	4275031	12787375	0.18 ± 0.01	0.05
890.8	C52 H92 N O8 P		20:0/24:6	29.95	2484232	312195	1579725	0.02 ± 0.01	0.01
892.8			x	31.35	1330663	726192	1513239	0.02 ± 0.01	0.01
894.8			x	21.001	1553124	449442	1136167	0.02 ± 0.00	0.01
898.8			x	24.305	541607	152237	620920	0.01 ± 0.00	0.00

902.8			x	31.857	689234	587942	1283029	0.02 ± 0.01	0.01
904.8			x	28.711	1036367	168661	892889	0.01 ± 0.00	0.00
904.8			x	33.228	844098	311795	1532716	0.02 ± 0.01	0.00
906.8			x	29.97	268066	166757	397731	0.01 ± 0.00	0.00
912.8			x	27.883	579315	230977	444169	0.01 ± 0.00	0.00
914.8			x	29.315	328246	145557	282196	0.01 ± 0.00	0.00
916.8	C54 H94 N O8 P	3.69	22:1/24:6	30.203	2310470	1024651	2842206	0.04 ± 0.01	0.01
918.8	C54 H96 N O8 P	2.25	22:1/24:5	31.556	2387515	94474	2526084	0.03 ± 0.02	0.01
922.8	C54 H100 N O8 P		22:0/24:4	23.863	1204663	726966	1575974	0.02 ± 0.01	0.01
924.8	C55 H90 N O8 P	5.07	46:3	25.382	2879109	821116	2846998	0.04 ± 0.00	0.01
926.8	C54 H104 N O8 P		22:1/24:1	26.539	642923	199166	559190	0.01 ± 0.00	0.00
Serine glycerophospholipids (PS)									
796.6	C44 H78 N O9 P		18:1a/20:4	23.529	766031	405481	1274968	0.24 ± 0.06	0.06
820.6	C46 H78 N O9 P		16:0p/24:6	24.216	1438357	31949	1414734	0.21 ± 0.16	0.05
822.6	C46 H80 N O9 P	7.86	16:1a/24:5	24.755	14569282	4937018	17750359	3.40 ± 0.17	0.82
822.6	C46 H80 N O9 P	0.67	16:0p/24:5	25.354	9384798	3714482	12235202	2.37 ± 0.26	0.57
824.6	C46 H82 N O9 P	5.52	16:0a/24:5	25.859	144768516	45872777	166301325	32.42 ± 0.85	7.85
834.6	C47 H80 N O9 P		17:0p/24:6	24.317	81527	31887	233958	0.03 ± 0.02	0.01
836.6	C47 H82 N O9 P	4.62	41:6a	25.69	5940519	552512	3257326	0.78 ± 0.48	0.19
836.6	C47 H82 N O9 P	4.36	17:0p/24:5	26.575	3805916	2042517	6499002	1.19 ± 0.30	0.29
838.6	C47 H84 N O9 P		17:0p/24:4	27.021	11294491	2749408	11921075	2.27 ± 0.32	0.55
848.6	C48 H82 N O9 P	1.25	18:1p/24:5	25.162	2940918	1609717	3430729	0.82 ± 0.26	0.20
848.6	C48 H82 N O9 P		18:1a/24:6	25.65	2255651	473749	2356642	0.43 ± 0.09	0.10
850.6	C48 H84 N O9 P	0.65	18:1a/24:5	26.261	52904440	11900821	35349053	9.02 ± 2.42	2.18
852.6	C48 H86 N O9 P	4.28	18:0a/24:5	28.094	46232344	15628357	54783892	10.69 ± 0.39	2.59
862.6	C48 H80 N O10 P		18:1/24:6	23.514	2470619	896448	3543599	0.63 ± 0.08	0.15
864.6	C48 H82 N O10 P		18:1/24:5	24.968	1512261	960981	2034640	0.47 ± 0.17	0.11
864.6	C48 H82 N O10 P	2.88	18:0/24:6	25.504	24673920	8300740	32337147	5.90 ± 0.53	1.43
864.6			x	26.041	2516518	62877	134790	0.21 ± 0.30	0.05
866.7	C48 H84 N O10 P	3.38	18:0/24:5	26.603	65127202	24172944	81588076	15.85 ± 1.23	3.84
888.6	C50 H84 N O10 P		20:2/24:6	24.319	1330825	453766	582835	0.24 ± 0.11	0.06
890.6	C50 H86 N O10 P	5.99	20:1/24:6	25.762	17169737	5264733	17378372	3.65 ± 0.16	0.88
892.6	C50 H86 N O10 P	2.46	20:1/24:5	26.944	21965041	7082939	23528169	4.83 ± 0.10	1.17
894.6	C50 H88 N O10 P		20:0/24:5	28.818	1697140	721374	2222632	0.44 ± 0.06	0.11
918.6	C52 H84 N O10 P		46:7	27.729	681309	342570	813444	0.18 ± 0.05	0.04
920.6	C52 H86 N O10 P		46:6	28.882	700628	102041	70690	0.08 ± 0.07	0.02
920.6	C52 H86 N O10 P		22:1/24:5	29.206	1150295	1010306	2643174	0.50 ± 0.22	0.12
920.6	C52 H86 N O10 P		46:6	29.616	654210	292300	210231	0.13 ± 0.08	0.03

936.6	C54 H82 N O10 P		24:6/24:6	23.212	7511341	2506948	7963282	1.67 ± 0.07	0.40
938.6	C54 H84 N O10 P	10.12	24:5/24:6	24.789	3500485	1453142	3947065	0.86 ± 0.13	0.21
940.6	C54 H86 N O10 P		48:10	25.951	1763323	781354	2357276	0.47 ± 0.08	0.11
944.6	C54 H90 N O10 P		48:8	28.775	80761	46837	0	0.02 ± 0.02	0.00
Inositol glycerophospholipids (PI)									
843.6	C45 H81 O12 P	10.79	16:0a/20:4	19.847	156225	20803	432404	0.84 ± 0.83	0.02
843.6	C45 H81 O12 P		15:0/20:4	20.05	125139	30063	83808	0.36 ± 0.06	0.01
855.6	C45 H77 O13 P	5.72	16:0/20:5	14.128	121755	47525	47866	0.37 ± 0.15	0.01
855.6	C45 H77 O13 P		x	20.221	45877	16110	65059	0.20 ± 0.06	0.01
857.6	C45 H79 O13 P	6.93	16:0/20:4	17.126	550361	77633	494212	1.57 ± 0.67	0.04
883.6	C47 H81 O13 P	5.65	18:0/20:5	19.67	317083	116828	203942	1.04 ± 0.19	0.03
885.6	C47 H83 O13 P	6.37	18:0/20:4	21.996	815776	444980	604718	3.28 ± 1.15	0.09
887.6	C47 H85 O13 P		18:0/20:3	24.976	132057	14155	29490	0.24 ± 0.18	0.01
891.6	C47 H89 O13 P		18:0/20:1	25.968	203949	92879	78673	0.66 ± 0.32	0.02
895.6	C49 H85 O12 P	5.92	16:0a/24:6	22.638	161062	60277	239409	0.72 ± 0.23	0.02
897.6	C49 H87 O12 P	5.41	16:0a/24:5	24.31	597842	68688	299670	1.32 ± 0.66	0.04
909.6	C49 H83 O13 P	3.46	16:0/24:6	20.324	1095523	263446	1072826	3.62 ± 0.85	0.10
909.6			x	20.746	132161	38894	54890	0.36 ± 0.12	0.01
911.6	C49 H85 O13 P	5.15	16:0/24:5	22.572	1874625	900704	2002774	7.97 ± 1.50	0.22
911.6	C51 H87 O13 P	8.54	18:0/22:5	23.636	226018	129009	122708	0.87 ± 0.42	0.02
913.6	C49 H87 O13 P	2.68	18:0/22:4	24.369	984729	168459	895833	2.92 ± 1.04	0.08
923.6	C51 H89 O12 P	6.61	17:0/24:6	24.636	309222	46163	81949	0.62 ± 0.38	0.02
925.6	C50 H87 O13 P	2.53	17:0/24:5	24.235	488775	37596	254405	1.03 ± 0.63	0.03
925.6	C51 H91 O12 P	1.79	x	26.423	95388	17902	110605	0.32 ± 0.14	0.01
935.6	C51 H85 O13 P	5.44	18:1/24:6	21.285	630036	231619	464411	2.15 ± 0.24	0.06
937.6	C51 H87 O13 P	3.57	18:1/24:5	23.215	662255	405488	507923	2.84 ± 1.17	0.08
937.6	C51 H87 O13 P	2.29	18:0/24:6	24.049	4648208	1467848	3588177	15.23 ± 0.49	0.43
939.6			x	24.657	118575	22887	40214	0.27 ± 0.12	0.01
939.6	C51 H89 O13 P	2.77	18:0/24:5	25.301	12605037	4346374	11106504	44.44 ± 1.57	1.24
939.6			x	26.294	43743	15258	36736	0.15 ± 0.00	0.00
953.6	C52 H91 O13 P	1.93	20:0a/24:5	26.266	327181	150238	178123	1.13 ± 0.41	0.03
963.6	C53 H89 O13 P	4.04	20:1/24:6	24.469	790784	129323	362500	1.84 ± 0.73	0.05
965.6	C53 H91 O13 P	4.61	20:1/24:5	25.564	804437	193490	616244	2.42 ± 0.38	0.07
967.6	C53 H93 O13 P		20:0/24:5	27.036	94843	14351	31570	0.20 ± 0.11	0.01
967.6			x	27.442	32080	5740	15678	0.08 ± 0.03	0.00
991.6	C55 H93 O13 P	11.39	22:2/24:5	26.205	141190	29566	33127	0.31 ± 0.17	0.01
993.6			x	28.413	66448	14391	15549	0.15 ± 0.08	0.00

Supplementary Figure S7. Examples of MS/MS spectra of polar lipids. Total lipids of the soft coral *Paragorgia arborea* were analyzed using reverse-phase liquid chromatography and tandem mass spectrometry at electrospray ionization (ESI) conditions in positive and negative modes. MS/MS spectra of the target ions, predicted structures, and product ions (*m/z*) are shown for several molecular species of ceramide aminoethylphosphonate (CAEP) and four glycerophospholipid (GPL) classes: ethanolamine, choline, inositol and serine GPL (abbreviated here as PE, PC, PI, and PS, respectively).

CAEP 20:3d/16:0, precursor ion $[M+H]^+$, *m/z* 669.5

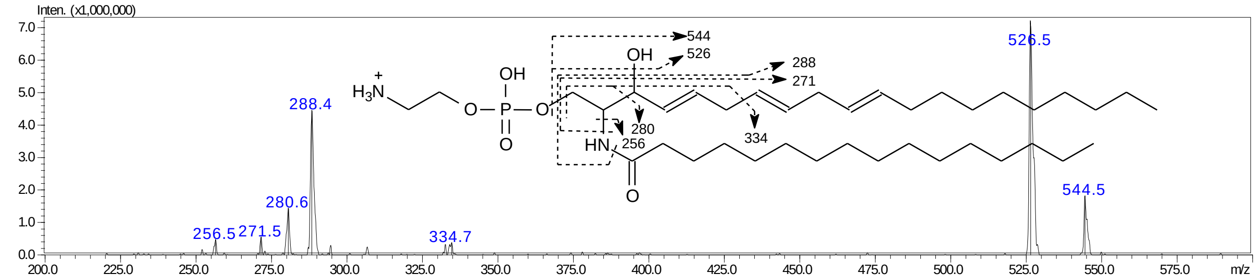

PC 16:0a/20:5, precursor ion $[M+Li]^+$, *m/z* 772.5

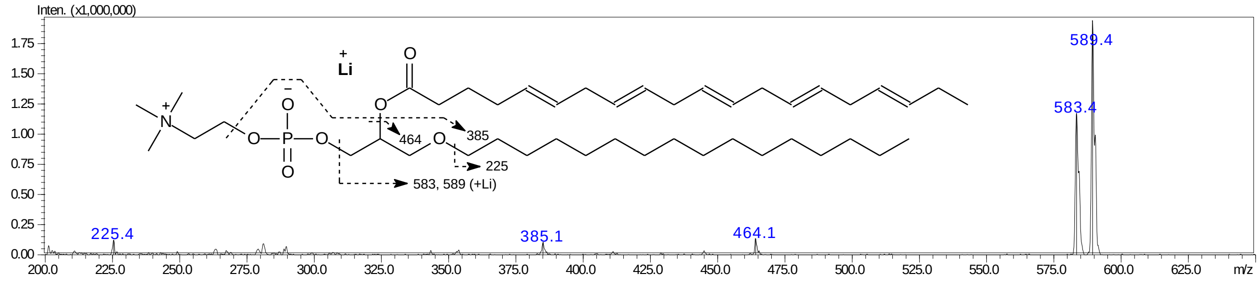

PC 16:0a/20:5, precursor ion $[M+HCOO]^-$, *m/z* 810.5

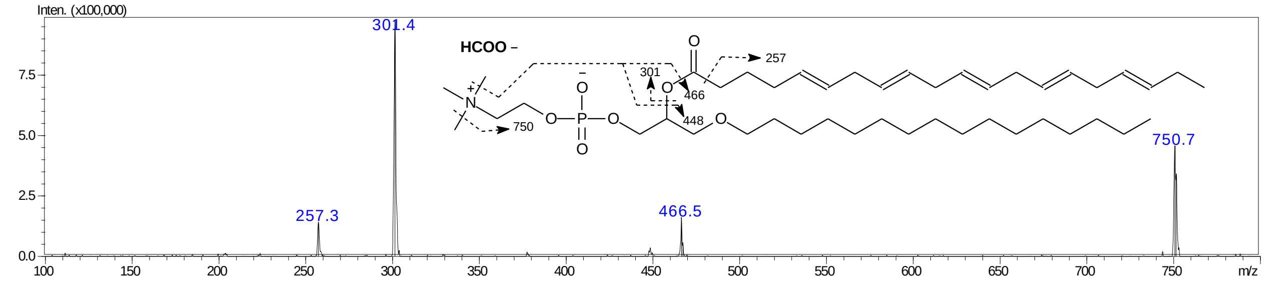

PC 16:0/20:5, precursor ion $[M+Li]^+$, *m/z* 786.5

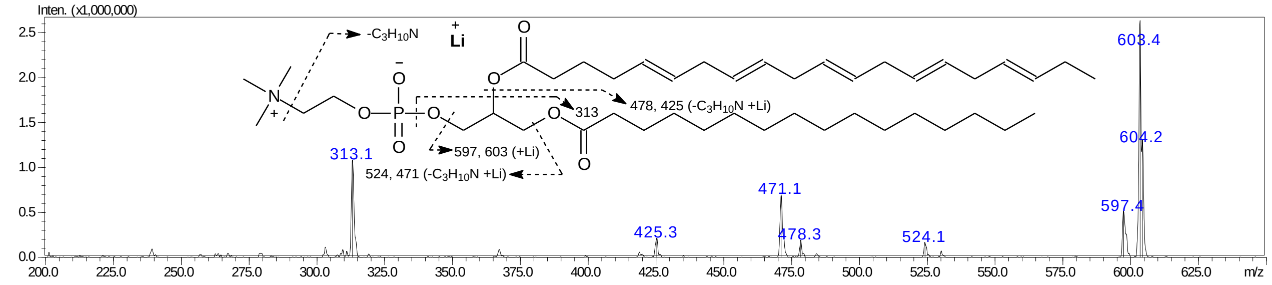

PC 16:0/20:5, precursor ion [M+HCOO]$^-$, m/z 824.5

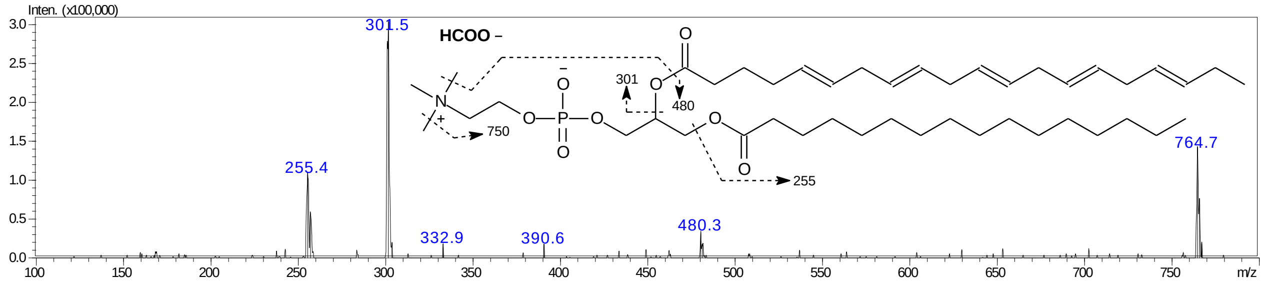

PE 16:1a/20:4, precursor ion [M+H]$^+$, *m/z* 724.5

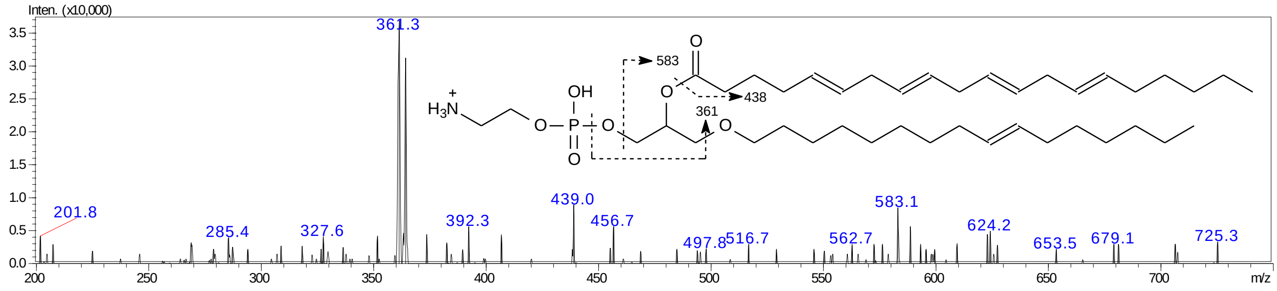

PE 16:1a/20:4, precursor ion [M–H]$^-$, *m/z* 722.5

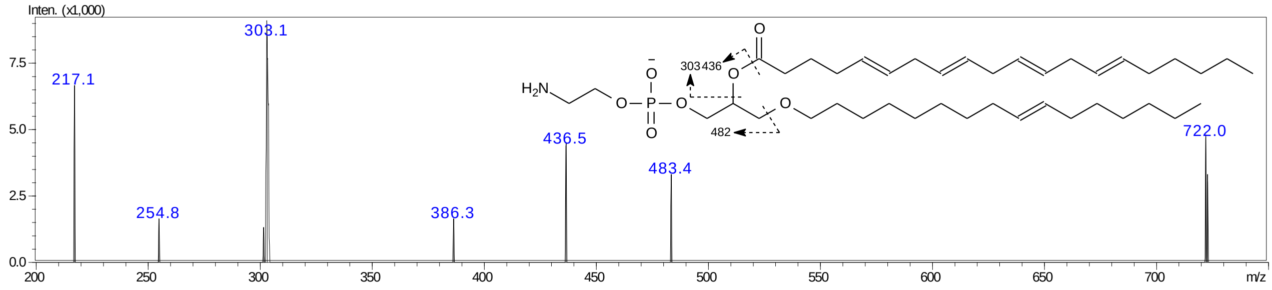

PE 16:0p/20:4, precursor ion [M+H]$^+$, *m/z* 724.5

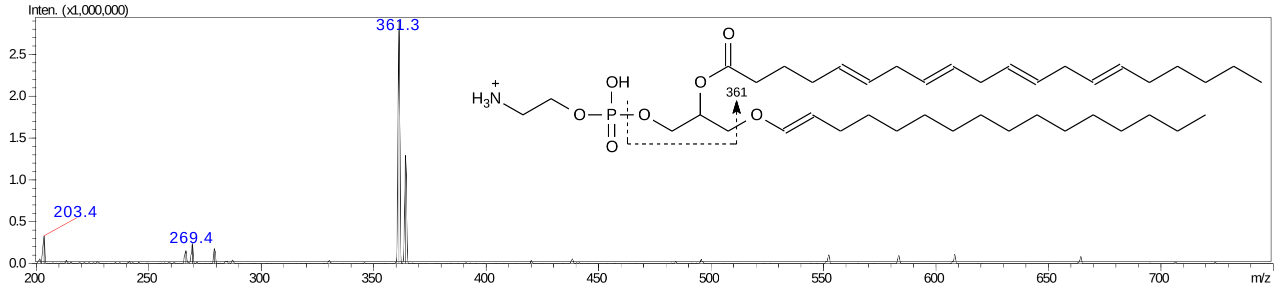

PE 16:0p/20:4, precursor ion [M–H]$^-$, *m/z* 722.5

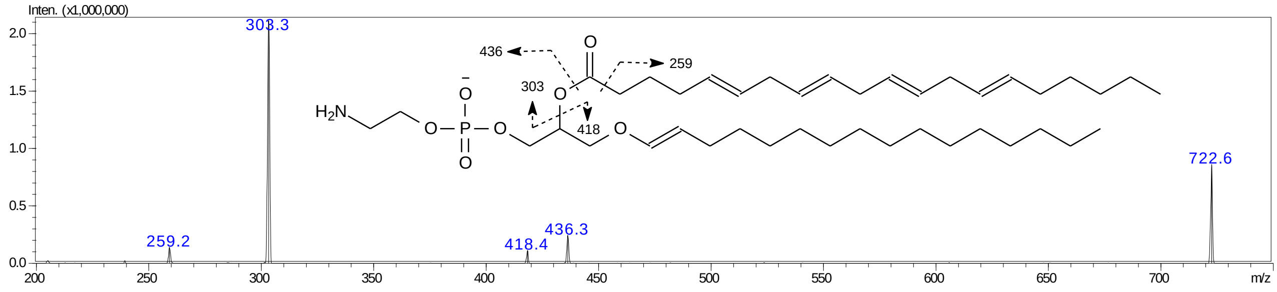

PS 16:0a/24:6, precursor ion [M+H]$^+$, *m/z* 822.6

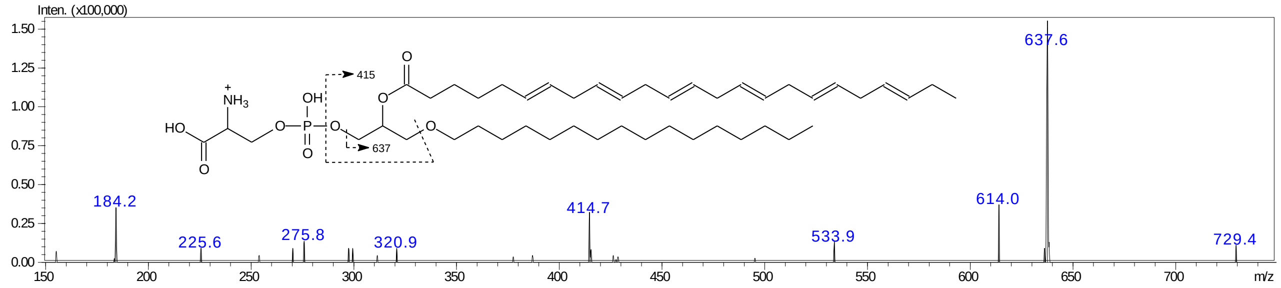

PS 16:0a/24:6, precursor ion [M–H]$^-$, *m/z* 820.6

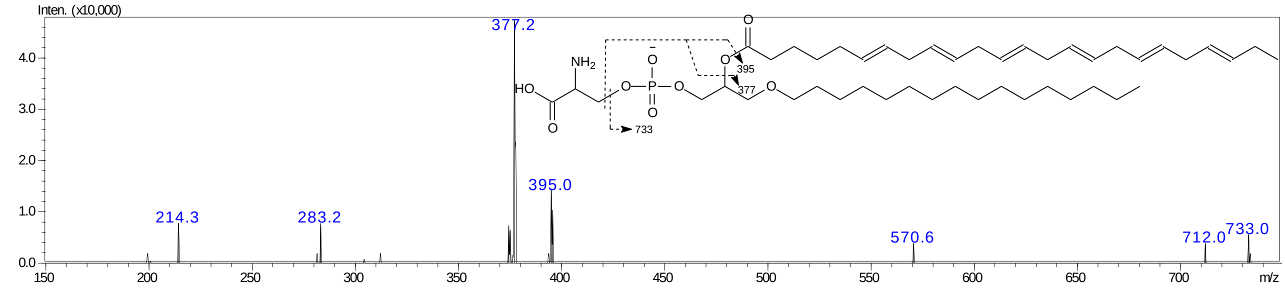

PS 16:0p/24:5, precursor ion [M+H]$^+$, *m/z* 822.6

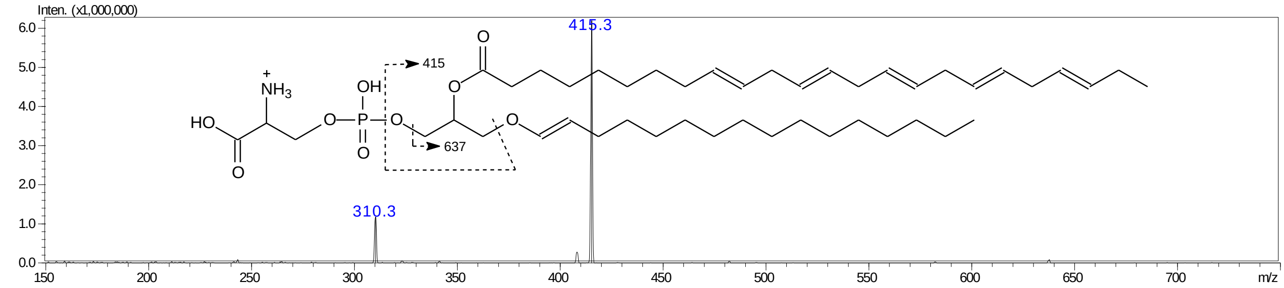

PS 16:0p/24:5, precursor ion [M–H]$^-$, *m/z* 820.6

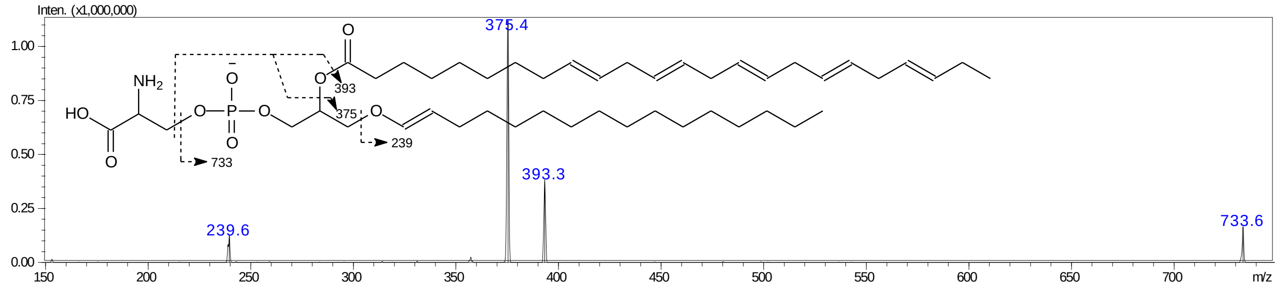

PS 18:0/24:5, precursor ion [M+H]$^+$, *m/z* 866.7

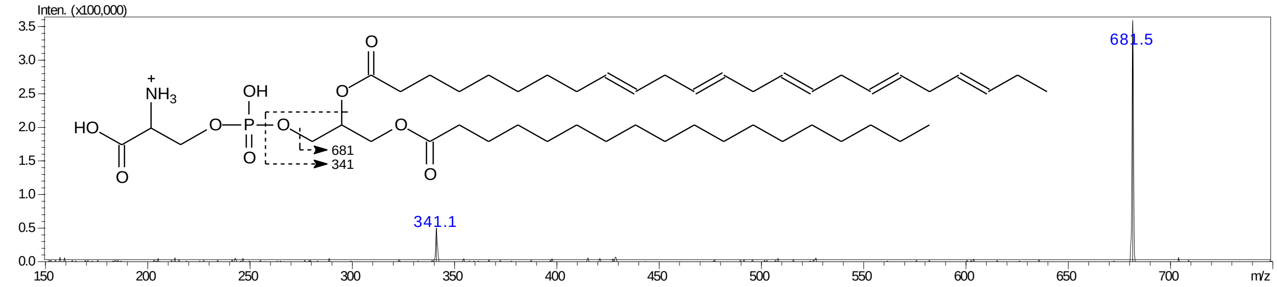

PS 18:0/24:5, precursor ion [M–H]$^{-}$, *m/z* 864.6

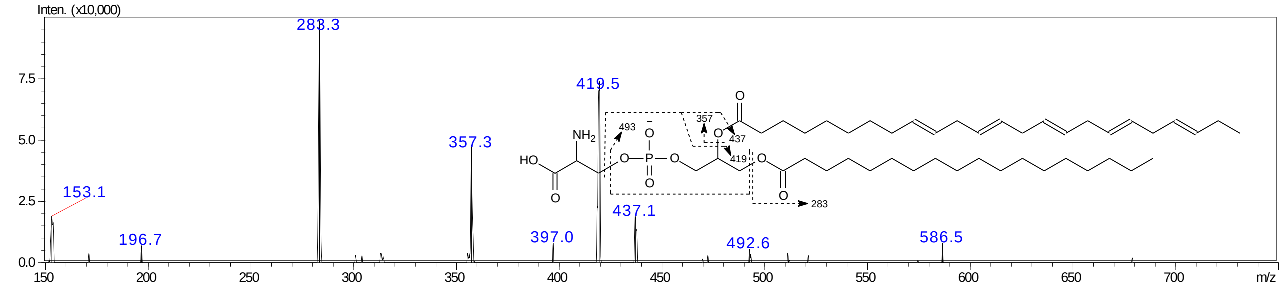

PI 16:0a/24:5, precursor ion [M–H]$^{-}$, *m/z* 897.6

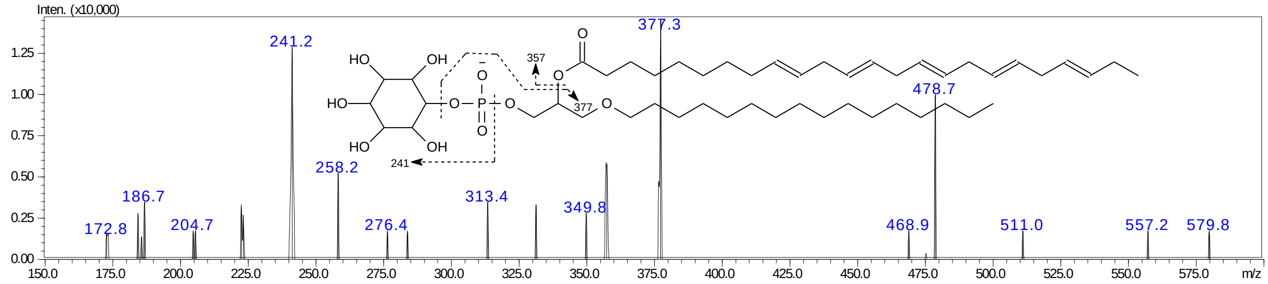

PI 16:0/24:5, precursor ion [M–H]$^{-}$, *m/z* 911.6

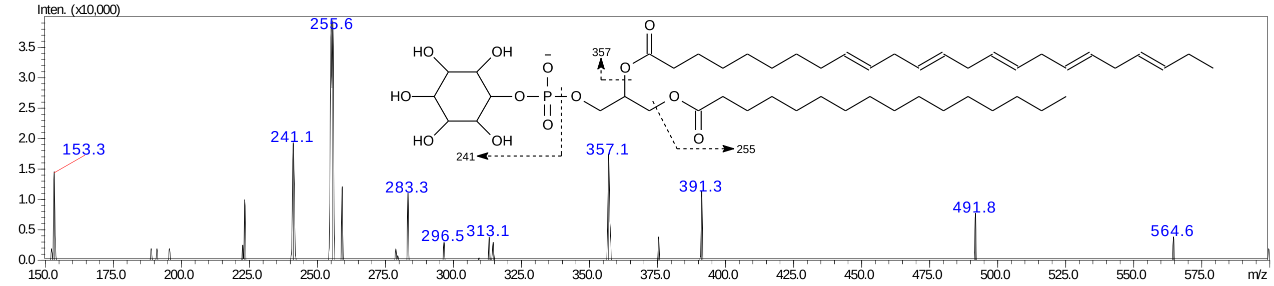

Supplementary Table S4. Non-polar parts of ether GPL and DAGE. A composition (% of lipid class, mean ± SD) of molecular species of diacylglycerol ethers (DAGE, n = 6) and ether glycerophospholipids (GPL, n = 3) with similar 1-*O*-alkyl-2-acyl parts in the soft coral *Paragorgia arborea*. Alkyl/acyl parts are abbreviated as Xa/Y; Xa is alkyl group and Y is acyl group (number of carbon atoms : number of double bonds) at positions *sn*-1 and *sn*-2 of a glycerol ether backbone. Ethanolamine, choline, inositol and serine GPL are abbreviated as PE, PC, PI, and PS, respectively. nd, not detected.

Alkyl and acyl groups in positions *sn*-1/*sn*-2	Content, % of lipid class				
	DAGE	PE	PC	PS	PI
14:0a/20:5	3.33 ± 1.02	nd	nd	nd	nd
14:0a/20:4	3.50 ± 1.00	nd	8.73 ± 0.73	nd	nd
16:0a/20:5	27.32 ± 1.85	0.67 ± 0.24	13.45 ± 1.87	nd	nd
16:0a/20:4+ 16:0p/20:4	33.17 ± 4.84	24.75 ± 1.30	29.73 ± 2.36	nd	0.84 ± 0.83
18:1a/20:4	1.24 ± 0.50	5.63 ± 0.22	5.65 ± 0.27	0.24 ± 0.06	nd
18:1a/20:5	3.22 ± 0.79	1.20 ± 0.22	2.97 ± 0.13	nd	nd
14:0a/24:5	1.99 ± 0.57	nd	nd	nd	nd
16:1a/24:5	nd	0.58 ± 0.10	0.14 ± 0.03	nd	nd
16:0a/24:6	9.93 ± 2.99	9.40 ± 0.15	2.59 ± 0.17	3.40 ± 0.17	0.72 ± 0.23
16:0a/24:5	4.18 ± 0.78	11.07 ± 0.93	1.7 ± 0.05	32.42 ± 0.85	1.32 ± 0.66
18:1a/24:6	1.24 ± 0.50	5.88 ± 0.65	0.36 ± 0.02	0.43 ± 0.09	nd
18:1a/24:5	nd	5.62 ± 0.28	0.18 ± 0.03	9.02 ± 2.42	nd
18:0a/24:5	nd	1.02 ± 0.22	0.14 ± 0.00	10.69 ± 0.39	nd
16:0/24:5	nd	nd	nd	nd	7.97 ± 1.50
18:0/24:6	nd	nd	0.37 ± 0.03	5.9 ± 0.53	15.23 ± 0.49
18:0/24:5	nd	0.35 ± 0.14	0.31 ± 0.03	15.85 ± 1.23	44.44 ± 1.57
